# Experimental study of TPU and SEBS additively manufactured leaflets for novel aortic valve polymer TAVI prosthesis

**DOI:** 10.3389/fbioe.2026.1959014

**Published:** 2026-10-21

**Authors:** Alex G. Kuchumov, Denis Khoroshev, Aleksandra Kutuzova, Aleksei Baranov, Oleg Krestyaninov, Ekaterina Barbashina, Pavel Zelenchuk, Edward Matjeka, Thanyani Pandelani, Fulufhelo Nemavhola

**Affiliations:** 1 Research Center for Genetics and Life Sciences, Sirius University of Science and Technology, Sirius, Russia; 2 Biofluids Laboratory, Perm National Research Polytechnic University, Perm, Russia; 3 Endovascular Surgery Department, E.N. Meshalkin National Medical Research Center, Novosibirsk, Russia; 4 Endovascular Surgery Department, Center of Surgery named after A. Vishnevsky, Moscow, Russia; 5 Department of Mechanical, Bioresources and Biomedical Engineering, School of Engineering, College of Science, Engineering and Technology, University of South Africa, Florida, South Africa; 6 Department of Mechanical Engineering, Faculty of Engineering and the Built Environment, Durban University of Technology, Durban, South Africa

**Keywords:** additive manufacturing, aortic valve, digital image correlation, polymeric heart valve (PHV), SEBS, TPU, uniaxial test

## Abstract

**Background and Objective:**

Transcatheter valve implantation is a cutting-edge technology for high and low risk patients with aortic stenosis. Bioprosthetic valves are limited due to several disadvantages as a long-term durability and structural valve degeneration. Polymeric aortic valve leaflet devices represent state-of-the-art technology to overcome limitations of bioprostheses. Nevertheless, more recent biocompatible and biostable polymers have revealed a significant potential. Additionally, 3D additive manufacturing opens new paths for patient-specific design of biomedical implants. Flexible polymers may not only mimic native aortic valve, but also may improve long-term durability. Polyurethanes and styrene polymers are promising materials for modern polymer valves. Nevertheless, mechanical properties of additive manufactured TPU or SEBS leaflets are not properly studied.

**Methods:**

Experimental study of TPU and SEBS samples and aortic valve leaflets for novel aortic valve polymer TAVI prosthesis is presented. Tensile tests and compression tests of three types of FDM-printed samples with various filament alignments (axial alignment, transversal alignment, diagonal alignment) were performed. The results of tests confirmed that longitudinal axial alignment achieve a maximum stress compared to transversal and diagonal directions. Compressive tests of TPU and SEBS 3D-manufactured aortic valve leaflets with digital image correlation technology were performed. Young’s modulus and Poisson’s ratio were evaluated. Moreover, parameters of constitutive models were found.

**Results:**

The TPU tensile specimen exhibits the highest stress/strain values at a speed of 100 mm/min, regardless of the filament alignment pattern. Slightly lower results are observed at a speed of 50 mm/min, which is likely associated with a wide range of plastic deformation. In the case of SEBS, a different pattern is observed: at 500 mm/min, it demonstrates high resistance to fracture, unlike TPU. A comparison of TPU and SEBS specimens at the same speeds and with the same alignment, based on maximum stress values, shows a minimal difference of 4% with the axial alignment at a speed of 50 mm/min; however, when comparing strains, the difference increases to 90%. The results of the compression experiment on aortic valve specimens made of TPU and SEBS at a speed of 30 mm/min showed that TPU force values for is considerably lower (4% of the maximum case), while for SEBS the spread increases to 20% of the maximum case.

**Conclusion:**

SEBS aortic valve leaflets were shown to be stiffer than TPU. It can be a step for choice of stiffer/softer leaflets for patient-specific device. The proposed approach can help manufacturers in elaboration and manufacturing of new TAVI polymer devices design made of TPU and SEBS polymers for clinical applications.

## 1 Introduction

Recently, transcatheter aortic valve implantation (TAVI) has become a standard, very successful technique for aortic valve pathology treatment (Chakos et al., 2017; Amin et al., 2025; Iannopollo et al., 2025; Nikas et al., 2025). TAVI’s indications have expanded as a result of numerous clinical trials confirming its safety and effectiveness (Lee et al., 2026). Patients at low surgical risk as well as those at high and intermediate surgical risk can now undergo the operation. Furthermore, TAVI treatments have already outnumbered surgical aortic valve replacements in a number of nations (Rosseel et al., 2023).

Traditional heart valve prostheses, whether mechanical or biological (xenopericardial), have a number of fundamental limitations: mechanical valves require lifelong anticoagulation, while biological valves are prone to calcification and structural degeneration within 10–15 years (David, 2021; Matjeka et al., 2026a; Matjeka et al., 2026b). Furthermore, the interaction between the TAVI stent frame and the valve leaflets that occurs during crimping and deployment contributes to accelerated structural degeneration (Kuchumov et al., 2025).

Polymeric heart valves (PHV) with flexible leaflets have the potential to overcome many of the previously described drawbacks of conventional treatment approaches (Singh et al., 2023). Contemporary biocompatible polymers demonstrate significant potential from biomechanical and biochemical point of view (Arif et al., 2019). Additionally, PHV manufacturing techniques enable high reproducibility and optimal cost (Rezvova et al., 2023). Several PHV TAVI devices like Foldax Tria (Kereiakes et al., 2021), Inflow (IHV Inc. - Catowice, Poland) (InFlow Valve-IHV-Innovations for Heart and Vessels, n.d), SAT (Strait Access Technologies Inc., Cape Town, South Africa) (Appa et al., 2022), PoliValve (Stasiak et al., 2020), Triskele (UCL Cardiovascular Engineering Laboratory) (Rahmani et al., 2017), PolyV-B (Rotman et al., 2019; Baylous et al., 2026) and some others were recently introduced. Some good reviews on polymer valves can be mentioned (Rotman et al., 2018; Singh et al., 2023; Wang et al., 2024; Mao Y. et al., 2025; Pil et al., 2026b).

In recent years, the treatment paradigm has been shifting toward tissue engineering and regenerative medicine, where additive manufacturing technologies play a key role, as 3D printing enables the fabrication of patient-specific implants with complex architectures and high precision (Coulter et al., 2019; Tschorn et al., 2022). This approach holds great promise for creating bioinspired constructs that replicate the multilayered structure of native heart valve tissue, which is critically important for restoring the natural biomechanics of the aortic root (Vernon et al., 2024).

Various materials like such as Styrene-Ethylene-Butylene-Styrene (SEBS) (Stasiak et al., 2020), Styrene-Ethylene-Propylene-Styrene (SEPS) (Fríes and Di Paolo, 2023), cross-linked poly(Styrene-block-IsoButylene-block-Styrene) (xSIBS) (Helbock et al., 2023), Polyhedral Oligomeric Silsesquioxane-Poly(Carbonate-Urea)Urethane (POSS-PCU) (Wang et al., 2024), and Thermoplastic Siloxane-PolyCarbonate Urethane (TSiPCU) (Hemalatha et al., 2024) are used for leaflets fabrication. SEBS and SEPS are two close thermoplastics materials (Maji and Naskar, 2022). Their main difference is in the structure of the “soft” block, which determines the scope of application. SEBS (with ethylene-butylene block) is stronger (Wang et al., 2025). SEPS (with an ethylene-propylene block) is famous for its exceptional transparency (Liu et al., 2020). xSIBS is an “improved version” of the well-known biocompatible polymer SIBS. Due to its cross-linking (this is indicated by the letter “x” - cross-linked), xSIBS has a unique resistance to destruction in the body and a low ability to cause thrombosis (Singh et al., 2023). POSS-PCU and TSiPCU: both materials are designed for long-term use as implants (Kidane et al., 2009). POSS-PCU is reinforced with special nanoparticles that give it exceptional strength and protect it from destruction (Tan et al., 2013). TSiPCU combines siloxane and polycarbonate components in its structure to achieve an optimal balance of flexibility, strength and biocompatibility, which is especially important for implants operating under load (Athanasopulu et al., 2019).

Comparison between novel polymer materials, traditional materials (ePTFE) and xenomaterials enables us to clarify why new polymers are of such interest in the field of developing new cardiovascular devices. New polymers (POSS-PCU, xSIBS, SiPUU) significantly exceed ePTFE in strength (Rezvova et al., 2023). Although ePTFE is the “gold standard” for vascular prostheses, it is too weak to create durable valve leaflets. Polymers were originally designed for cyclic loads (opening/closing). They possess elasticity close to that of native tissue (elongation of 500%–700%) and high fatigue strength (Vinogradov et al., 2001), while ePTFE for valve leaflets is a compromise, non-optimal material (Bui et al., 2021). The main advantage of new polymers over xenomaterials (bovine/porcine pericardium) is the combination of high strength and the absence of calcification.

(Yu et al., 2025) Studies of native human aortic valve tissue show that it is anisotropic (its properties depend on the direction of load) and has a complex layered structure (Anssari-Benam et al., 2011; Matjeka et al., 2026b). Contemporary bioengineering strives to reproduce this complexity in polymers (for example, TSiPCU with controlled segment distribution) to create an ideal prosthesis (Chaudhari et al., 2025).

Nevertheless, there are some limitations with these materials dealing with complexity with fused deposition modeling (FDM) fabrication. Furthermore, some materials are commercial and their production is limited. But on the basis of review, it was shown that polyurethanes and styrene polymers are most perspective materials for aortic valve leaflets 3D fabrication. So, thermoplastic polyurethane (TPU) и styrenic block copolymer (SEBS) (Maji and Naskar, 2022) are appropriate.

It should be noticed that mechanical properties studies of these materials in form of filaments for FDM 3D printing and 3D printed aortic valve leaflets are rare. While several studies have thoroughly characterized mechanical behavior of these polymers as cast films (Sakurai et al., 1999) or molded sheets (Wang et al., 2021), data on their performance in the filament form required for fused deposition modeling (FDM) 3D printing is significantly lacking. This gap is critical because the FDM extrusion process subjects the polymer to thermal and shear stresses that can alter its molecular orientation, crystallinity, and consequently its final mechanical properties. Furthermore, even fewer studies have evaluated the properties of complete 3D printed aortic valve leaflets, where the layer-by-layer deposition introduces anisotropy and potential interlayer adhesion weaknesses not present in bulk material. The limited research available suggests that printed leaflets may exhibit different failure modes and fatigue resistance compared to their cast analogues, yet systematic data remains sparse. Therefore, future work must prioritize the characterization of FDM-printed filaments and leaflet structures under physiologically relevant cyclic loading to validate these materials for transcatheter aortic valve implantation (TAVI) applications.

Constitutive models of TPU and SEBS are necessary to develop for further fluid-structure interaction analysis of novel biopolymer TAVI device. These models serve as the mathematical foundation that describes how these polymeric materials mechanically respond to the complex, multi-axial loads experienced during cardiac cycling. Without accurate constitutive descriptions, numerical simulations of the valve’s opening and closing behavior—particularly the interaction between blood flow and the deforming leaflets—will lack predictive reliability. The hyperelastic and viscoelastic nature of TPU and SEBS requires sophisticated constitutive frameworks capable of capturing large deformations and rate-dependent responses under pulsatile flow conditions. Developing such models is particularly critical for TAVI devices. Ultimately, validated constitutive models will enable virtual testing of novel biopolymer TAVI prostheses, significantly accelerating the design optimization process while reducing the need for costly and time-consuming experimental iterations.

The manuscript is arranged as follows. Section 2 describes tensile and compression tests of TPU/SEBS with three filament alignments. Experimental study of 3-D printed aortic valve leaflets compression is also presented. Additionally, this section contains information about digital image correlation technique for TPU/SEBS aortic valve leaflets compression. Section 3 presents the results of experimental studies. Section 4 contains the obtained results, which are discussed and compared with other studies. The study’s limitations and future directions are also considered.

## 2 Methods

We propose a comprehensive study of the mechanical properties of polyurethane-based and styrene block copolymer materials. The first part of the study involved uniaxial and compression tests of samples. Next, TPU and SEBS aortic valve leaflets were manufactured and compressed. The second part of the study involved determination parameters of tested materials and finite element analysis of aortic valve leaflets compression.

### 2.1 Additive manufacturing of dog bone samples TPU/SEBS with three filament alignments for tensile tests

TPU and SEBS filaments with a diameter of 1.75 ± 0.05 mm were used as materials for FDM 3D fabrication. TPU filament (TPU-87A, Shenzhen ESUN Industrial Co. Ltd, Shenzhen, China) and SEBS filament (Filaflex SEBS, Recreus Industries S.L., Alicante, Spain) were utilized. 3D models of dogbone samples were created using software Blender (Blender Foundation, Netherlands). Figure 1 exhibits the geometry of tensile test specimens designed according to the ISO 527-2:2012. The overall length of the dogbone sample was 140 mm and its thickness was 2 ± 0.2 mm (other dimensions are shown in Figure 1d). Table 1 contains data for TPU and SEBS filaments.

**FIGURE 1:**
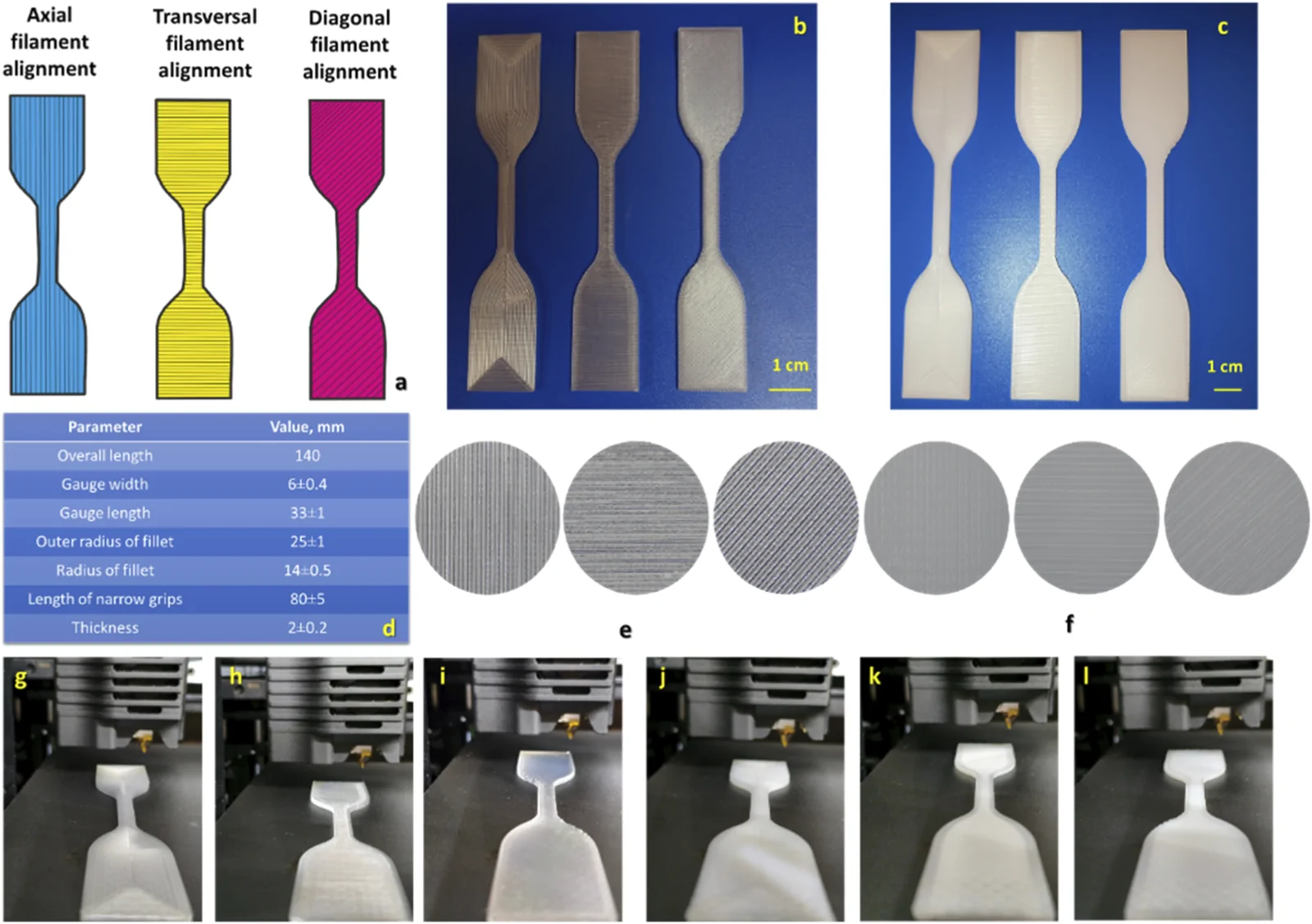
Dimensions of TPU and SEBS dogbone samples for uniaxial tensile tests: **(a)** – scheme of longitudinal, perpendicular and diagonal filament alignments, respectively, **(b)** – TPU samples with various filament orientation, **(c)** – SEBS samples with various filament orientation **(d)** – table with samples dimensions, **(e)** – filament alignments on printed TPU samples, **(f)** – filament alignments on printed SEBS samples, **(g)** – FDM printed TPU sample with longitudinal filament alignment, **(h)** – FDM printed TPU sample with perpendicular filament alignment, **(i)** – FDM printed TPU sample with diagonal filament alignment, **(j)** – FDM printed SEBS sample with longitudinal filament alignment, **(k)** – FDM printed SEBS sample with perpendicular filament alignment, **(l)** – FDM printed SEBS sample with diagonal filament alignment.

**TABLE 1:** TPU and SEBS data.

Characteristics	Test method	TPU	SEBS
Density (g/cm^3^)	ISO 1183	1.1–1.25	0.94–0.98
Shore hardness	ISO 868	60–90 A	70 A
Rebound resilience (%)	ISO 1133	30%–70%	45%–75%

Three types of samples with transversal (8 TPU and 8 SEBS), axial (8 TPU and 8 SEBS) and diagonal (8 TPU and 8 SEBS) filament alignments were created (Figure 1). STL models of dogbone bone samples were imported into Raise3D IdeaMaker® free slicing software (Raise 3D Technologies, Inc., Irvine, United States). The nozzle diameter was 0.4 mm, the layer height was 0.2 mm, and the extrusion temperature was 233 °С and 255 °С for TPU and SEBS, respectively. The settings were selected on the basis of preliminary test prints with selected materials. As a result, the control program (g-code) for printing was formed. The parts were printed without support structures to ensure sample flatness and high surface quality. The printing angle for samples with transversal and axial directions was 90° and 0°, respectively. For samples with a diagonal orientation, the printing angle was +45°. Time of FDM printing of each TPU sample was 79 min, while each SEBS sample was printed for 44 min.

The extrusion width for TPU was 0.3 mm, while for SEBS this value was 0.4 mm. The temperature of bed for printing the first layer of TPU was 50 °C. The temperature of bed for printing was 80 °C. The printing speed for the TPU dogbone sample was 20 mm/s (the first three layers were printed at a speed of 15 mm/s). The printing speed for SEBS dogbone sample was 40 mm/s (the first three layers were printed at a speed of 10 mm/s). The printing options of TPU and SEBS dogbone samples are presented in Table 2.

**TABLE 2:** FDM printing options of TPU and SEBS dogbone samples for tensile test.

Material	Thermal, °С		Speed, mm/s	Geometric, mm		
	Bed temperature	Nozzle temperature		Layer height	Nozzle diameter	Extrusion width
TPU	50	233	20	0.2	0.4	0.3
SEBS	80	255	40	0.2	0.4	0.4

### 2.2 Additive manufacturing of samples TPU/SEBS for compression tests

Samples for compression were designed according to the ISO 604:2002 standard with a cuboid shape; their dimensions are presented in Table 3.

**TABLE 3:** Dimensions TPU and SEBS samples for compression test.

Parameter	Value, mm
Length	10 ± 0.2
Width	10 ± 0.2
Thickness	10 ± 0.2

The nozzle diameter was 0.4 mm, the layer height was 0.14 and 0.2 mm, and the extrusion temperature was 233 °С and 265 °С for TPU and SEBS, respectively. The extrusion width for TPU was 0.2 mm, while for SEBS this value was 0.4 mm. The temperature of bed for printing TPU sample was 50 °C. The temperature of bed for printing was 75 °C. The printing speed for the TPU and SEBS samples was 15 mm/s.

The same three filament alignments were used in printing process (18 TPU and 18 SEBS).

FDM manufacturing of samples with axial alignment was used by application “inner shell” setting. In slicer software it is necessary to open a window “Prepare to slice” and after that select a button “Select template” and after that “Advanced settings.” At tab “Layer,” parameter “Shells” was set as 100, parameter “First layer solid fill pattern type”: Concentric and parameter “First layer shells”: 100. These parameters regulate inner fill of a sample. After that it is necessary to go to “Solid fill” tab and disable parameters “Base solid fill layers,” “Solid fill layers,” “Top surface solid fill layers,” “Bottom surface solid fill layers.”

FDM-printing of samples with diagonal alignment (+45) was performed with Solid fill settings. The parameters values are set in “Advanced settings” window. At “Layer” tab, “First layer shells,” “Shells” set as 0. After that it is necessary to go to “Solid fill” tab and set parameters “Base solid fill layers” – 100, “Solid fill layers” – 100, “Top surface solid fill layers” – 0, “Bottom surface solid fill layers” – 1. An angle of solid fill is set at tab “Top and down solid fill angle,” parameter “Angle” is set as 45. The printing settings for transversal filament alignment samples are similar to the printing settings for diagonal samples. All the same steps are being done. The difference is in the last angle parameter, the value is 0. After printing, the samples were kept under special conditions according to ISO 291:2008 (a standard atmosphere 23/50 (with air temperature of 23 °C and a relative humidity of 50%). All the samples were marked, and their exact dimensions were measured. FDM printing options of TPU and SEBS samples for compression tests are presented in Table 4.

**TABLE 4:** FDM printing options of TPU and SEBS samples for compression tests.

Material	Thermal, °С		Speed, mm/s	Geometric, mm		
	Bed temperature	Nozzle temperature		Layer height	Nozzle diameter	Extrusion width
TPU	50	233	15	0.14	0.4	0.2
SEBS	75	265	15	0.2	0.4	0.4

### 2.3 Additive manufacturing of TPU/SEBS aortic valve leaflets samples

The parameterized model of the aortic valve was created in the COMSOL Multiphysics (Comsol Inc., Stockholm, Sweeden), using a specialized internal program based on the analysis of application builder models. The geometric parameters of the model under study are shown in Figure 2. The nozzle diameter was 0.2 mm, the layer height was 0.14 mm, the extrusion width was 0.2 mm, and the extrusion temperature was 233 °С and 265 °С for TPU and SEBS, respectively. The bed temperature for printing of TPU was 50 °C. The temperature of bed for printing was 65 °C.

**FIGURE 2:**
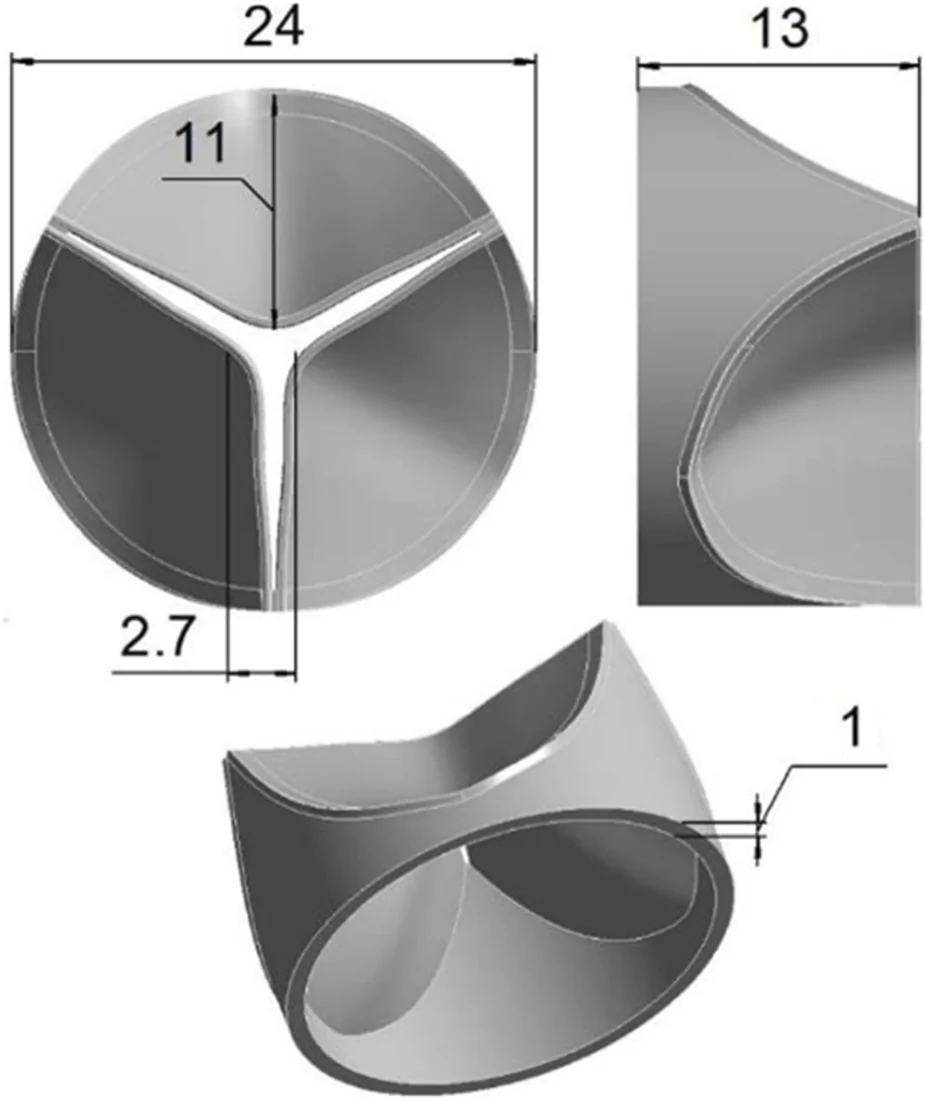
Geometry parameters and dimensions of aortic valve model.

The printing speed for the TPU aortic valve leaflets sample was 20 mm/s (the first three layers were printed at a speed of 15 mm/s). The printing speed for SEBS aortic valve leaflets sample was 15 mm/s. The cooling of the TPU sample on the first two layers was 25%, and then it was set to a maximum of 100%. The cooling of the SEBS sample on the first layer was 70%, and then it was set to 100%. Time of FDM printing of each TPU sample was 32 min, while each SEBS sample was printed for 35 min. The printing options of TPU and SEBS aortic valve leaflets samples are presented in Table 5. In total, 6 TPU samples and 6 SEBS samples were printed.

**TABLE 5:** FDM printing options of aortic valve lealets samples for TPU and SEBS.

Material	Thermal, °С		Speed, mm/s	Geometric, mm		
	Bed temperature	Nozzle temperature		Layer height	Nozzle diameter	Extrusion width
TPU	50	233	20	0.14	0.2	0.2
SEBS	65	265	15	0.14	0.2	0.2

### 2.4 Tensile tests of TPU/SEBS samples with DIC

To determine the mechanical properties of TPU and SEBS and their compliance with native aortic valve leaflets, a uniaxial tensile test was conducted using a universal testing machine STU-115 (Inteltest, Moscow, Russia) at a constant strain rate and a temperature of 23 °C. Each dogbone specimen was clamped in the grips of the testing machine with a load cell of 1 kN. Dogbone samples were fixed with pneumatic side-action grips to avoid a slip effect (Figure 3a). Next, a testing program was set based on specimen elongation (crosshead displacement). Local engineering strain was calculated from the DIC-measured displacements within the gauge section, using a reference length of approximately 20 mm. This approach avoids errors from machine compliance and grip slippage. Additionally, the dependence of the strength characteristics of the tested material on the loading rate of the specimens (50, 100, and 500 mm/min) was studied. TPU and SEBS samples with three types of filament alignment were tested with various loading rates. The criterion for the test elimination was the sample rupture. For TPU samples with axial filament alignment, an additional criterion of test elimination was adopted: the maximum displacement of 110 mm related to restrictions on lengths of hoses for pneumatic side clamps. After tests, it was concluded to perform tensile test with 100 mm/min loading rate. Thus, tensile tests were carried out for the remaining dogbone samples (30 samples) with the chosen speed. All samples also were marked and tested by using the DIC (digital image correlation) system RUDIC (Inteltest, Moscow, Russia). Based on the test results, the elastic modulus in the linear region of the stress–strain curve was determined, as well as the yield strength, maximum stress, strain at maximum stress, and maximum plastic strain.

**FIGURE 3:**
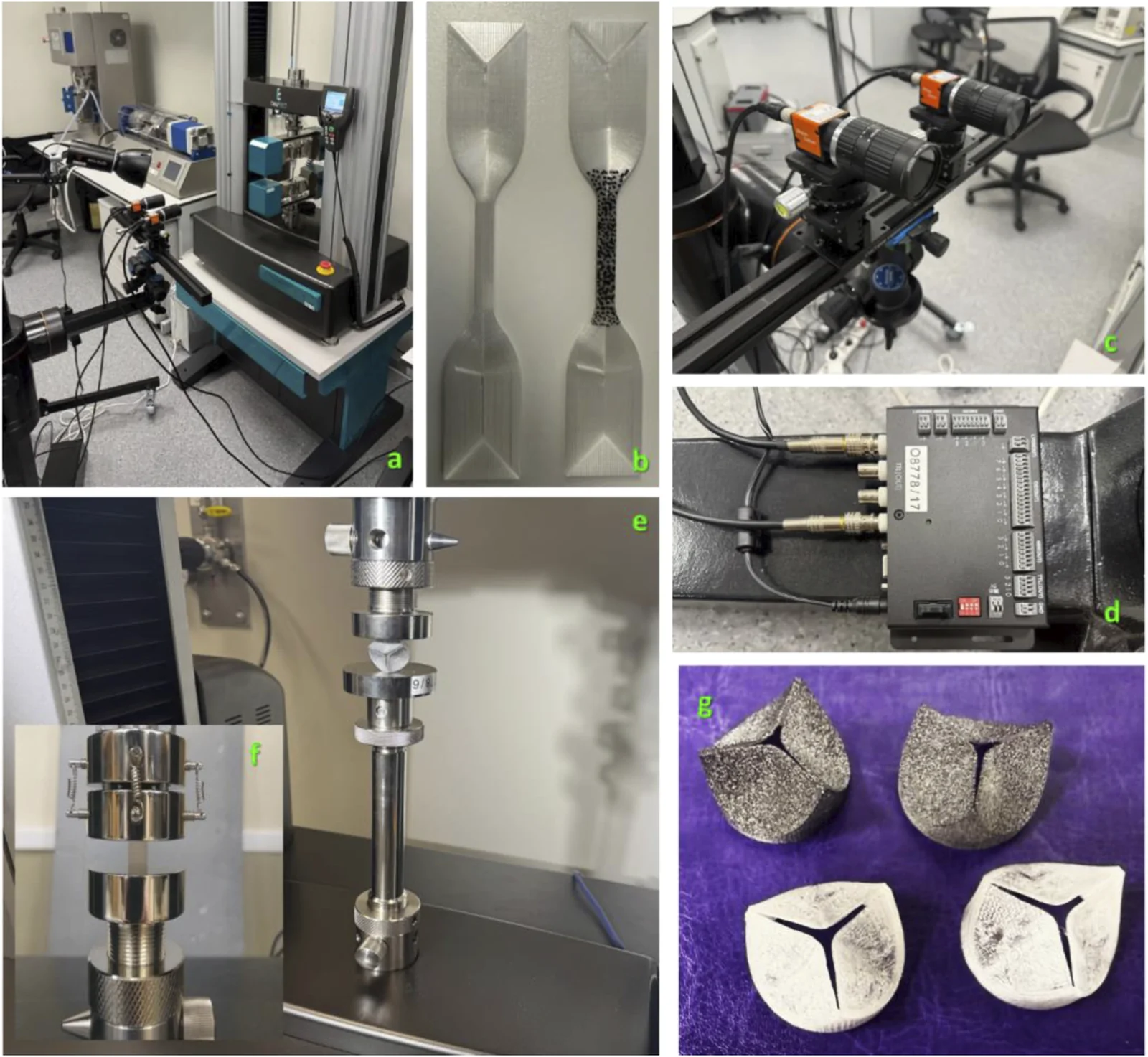
Experiments for TPU and SEBS samples: **(a)** – TPU sample in uniaxial testing machine, **(b)** – TPU sample without/with marks for DIC analysis, **(c)** – cameras, **(d)** – DIC synchronizer, **(e)** – TPU aortic valve leaflets under compression, **(f)** – TPU sample for compression test, **(g)** – TPU aortic valve leaflets samples without/with marks for DIC analysis.

Prior to testing, a random speckle pattern was applied to the gauge section of the specimens using commercial matte acrylic white and black primers (KUDO, Moscow, Russia). To minimize chemical interaction and potential degradation of the leaflet materials, the paint was applied by spraying in several thin, uneven layers from a distance of approximately 30 cm, which facilitated rapid solvent evaporation. The deformation process was recorded using a non-contact optical method with two monochrome cameras Mars5000S-35UM (Hangzhou Vision Datum Technology Co., Ltd., Hangzhou, China) with a resolution of 2,448 × 2,048 pixels and a maximum frame rate of 35 fps. Surface illumination was provided by an LED lamp Jinbei EFD-60M (COB source, 60 W, 5600 K, CRI >95) to ensure stable high-contrast imaging without thermal effects. Optical calibration of the stereo system was performed using a flat target with a regular dot grid; a set of images acquired at various orientations was processed in the RUDIC software environment, which implements a digital image correlation (DIC) algorithm. This technique allowed not only to determine the averaged elastic constants, but also to calculate Poisson’s ratio for each specimen type, revealing differences in transversal contraction depending on the structural orientation of the polymer chains.

### 2.5 Compression tests of TPU/SEBS samples with DIC

TPU and SEBS specimens were placed between two parallel rigid plates of the testing machine (Figure 3f). The plates were placed strictly parallel to ensure a uniform load distribution on the specimen. Next, the upper plate was gradually moved downwards to apply a compressive force to the specimen. The rate of compression was 1 mm/min. As the specimen was compressed, its reduction in height was measured using displacement gauges and DIC technique. The stress-strain relationship in the form of a compression diagram was recorded. The obtained data were analyzed to evaluate the strength and deformation properties of TPU and SEBS.

### 2.6 Compression tests of TPU/SEBS aortic valve leaflets samples with DIC

A sample of aortic valve leaflets was installed between two rigid plates on the side surface as shown in Figure 4. A square 15 mm^2^ × 15 mm^2^ double-sided adhesive tape backing was glued to the lower rigid plate, then a sample of aortic valve leaflets was installed on it. This ensured that the sample was fixed to the compression rig. Next, the upper plate was gradually moved downwards to apply a compressive force to the specimen. The rate of compression was 30 mm/min. The experiment stopped when the displacement of the upper plate reached 10 mm. Thus, the force-displacement curves are obtained.

**FIGURE 4:**
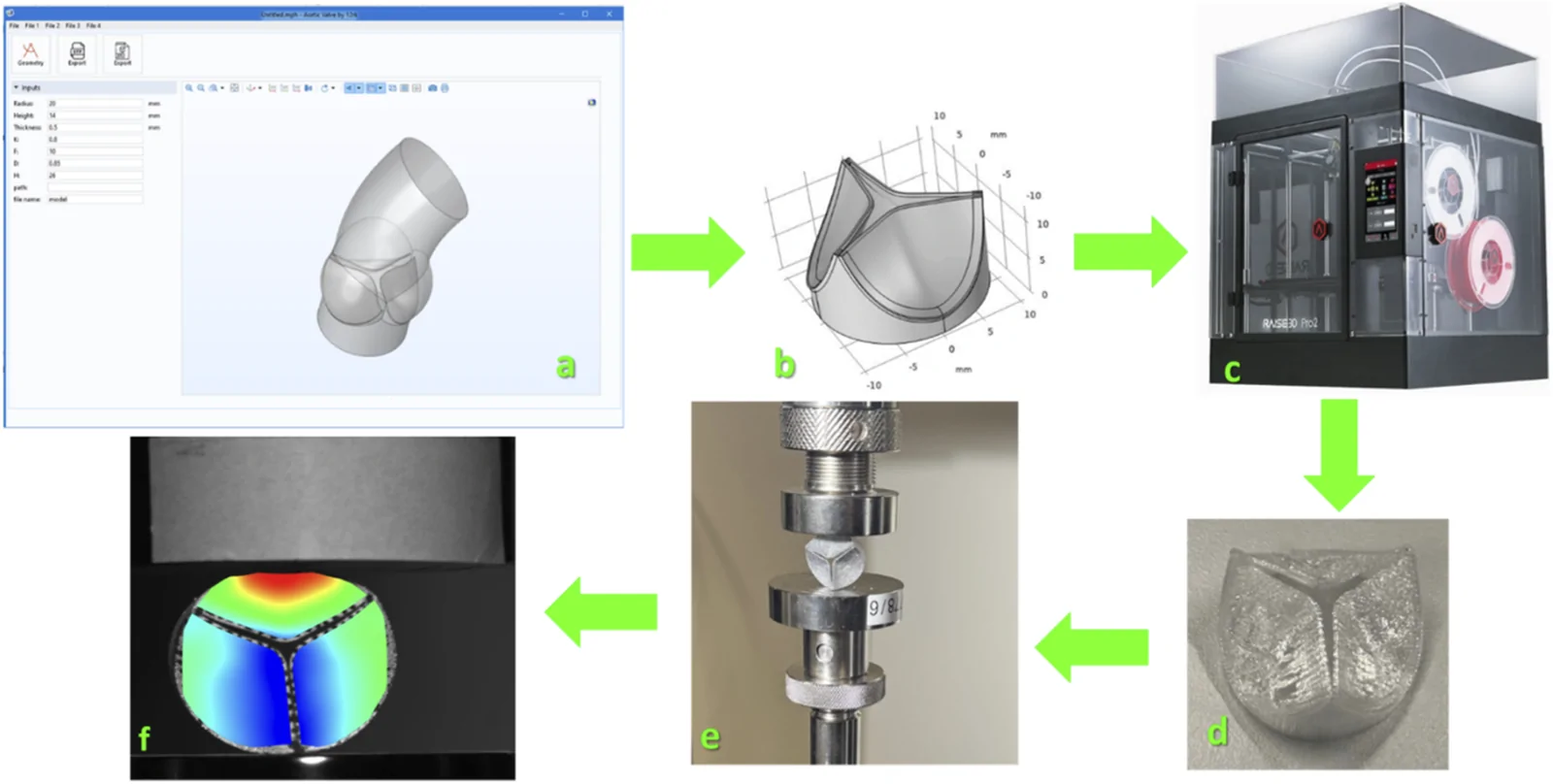
Pipeline for TPU/SEBS aortic valve leaflets compression test: **(a)** – 3D geometry design, **(b)** – aortic valve 3D model, **(c)** – FDM printing, **(d)** – FDM-manufactured sample, **(e)** – aortic valve leaflets sample under compression, **(f)** – DIC image of aortic valve sample compression.

Full-field displacement and strain measurements were obtained using a three-dimensional digital image correlation system RUDIC (Inteltest, Moscow, Russia). Prior to testing, each specimen was prepared with a high-contrast stochastic speckle pattern to enable accurate surface tracking. A uniform white base coat was first applied to the specimen surface using aerosol spray, followed by the application of randomly distributed black speckles by black aerosol spray. The speckle pattern quality was verified by ensuring a 50% grayscale distribution and adequate speckle density for the chosen field of view.

DIC system consisted of two synchronized CCD cameras mounted on a rigid stereo bracket. The cameras were positioned at a stereo angle of approximately 25° and focused on the frontal surface of the specimens. The distance from the cameras to the specimen surface was set to 300 mm, yielding a spatial resolution of approximately 25 μm/pixel. Image pairs were acquired at a frame rate of 30 Hz. Strain fields were derived from the displacement data using a Lagrangian finite strain formulation. The measurement accuracy of the DIC system was validated prior to testing by comparing DIC-derived displacements with calibrated images, yielding a discrepancy of less than 0.5% over a displacement range of 5 mm. Synchronization between the universal testing machine and the DIC system was achieved by triggering image acquisition at the onset of crosshead movement. Force, displacement, and time data from the testing machine were exported and temporally aligned with the DIC strain data. This procedure enabled the direct correlation of global stress-strain curves with local strain distributions obtained from DIC, facilitating the identification of strain localization, heterogeneity, and failure initiation sites across different material compositions and filament alignment configurations.

### 2.7 Constitutive models parameters estimation

To enable accurate finite element simulations of the polymeric aortic valve leaflets, it is essential to calibrate hyperelastic constitutive models for both TPU and SEBS. While the experimental uniaxial tensile data presented in Section 2.4 provide the raw material response, the finite element analysis requires these data to be expressed as material constants within a constitutive model. The Mooney-Rivlin hyperelastic model is widely used for elastomeric and thermoplastic polymers, as it captures the nonlinear stress–strain behaviour observed in TPU and SEBS up to large deformations (Huri, 2024). This model was chosen due to its high fidelity (it allows correctly describe a non-linear behavior to 100%–200%), flexibility (additional parameters can describe complex segments of stress-strain curve) and simplicity.

Estimation of Mooney-Rivlin parameters was performed by using the curve-fitting tools in the ANSYS Workbench Engineering Data module. The workflow is applicable to both the 5-parameter Mooney-Rivlin model for TPU (which exhibits more complex rate-dependent and anisotropic behaviour due to FDM printing) and the 2-parameter Mooney-Rivlin model for SEBS (which is adequately described by a simpler formulation).

The Mooney-Rivlin strain energy potential is expressed as a polynomial function of the first and second invariants of the right Cauchy-Green deformation tensor

$$W=C_{10}\left(I_{1}-3\right)+C_{01}\left(I_{2}-3\right)+{C_{20}\left(I_{1}-3\right)}^{2}+C_{11}\left(I_{1}-3\right)\left(I_{2}-3\right)+{C_{02}\left(I_{2}-3\right)}^{2}+\frac{1}{d}{\left(J-1\right)}^{2},$$ (1)

where *W* is the strain energy density, *I*_1_ and *I*_2_ are the first and second invariants of the right Cauchy-Green deformation tensor, *C*_10_, *C*_01_, *C*_20_ *C*_11_ *C*_02_ are material constants, *d* is the incompressibility parameter (typically set to a small value for nearly incompressible behaviour), *J* is the Jacobian determinant of the deformation gradient.

For the 2-parameter model (used for SEBS), the higher-order terms (*C*_20_, *C*_11_, *C*_02_) are omitted, and the potential reduces to:

$$W=C_{10}\left(I_{1}-3\right)+C_{01}\left(I_{2}-3\right)+\frac{1}{d}{\left(J-1\right)}^{2}.$$ (2)

The material constants are obtained by fitting the experimental stress–strain data to these (Equations 1, 2) using a least-squares minimization algorithm within ANSYS Curve Fitting tool. An algorithm of curve fitting in ANSYS is presented in Supplementary Material 1. Mooney-Rivlin model constants describing non-linear behavior of TPU and SEBS samples are presented in Table 6.

**TABLE 6:** Mooney-Rivlin model constants describing non-linear behavior of TPU and SEBS samples.

Plastics	Constitutive model	Constants
TPU	5 parameter Mooney–Rivlin model	*C*_01_ = −14.2 MPa, *С*_10_ = 18.14 MPa, *С*_11_ = −97.4 MPa, *С*_02_ = 60 MPa, *С*_20_ = 35.09 MPa
SEBS	2 parameter Mooney–Rivlin model	*С*_01_ = −68.1 MPa, *С*_10_ = 85.9 MPa

### 2.8 Finite element simulation of TPU/SEBS aortic valve leaflets compression

Compression modeling of the aortic valve was performed in ANSYS Workbench 2021 R2 (Ansys Inc., Cannonburg, United States). The model consists of the aortic valve and two rigid plates, as shown in Figure 5. The two perfectly rigid plates are represented as parallelepipeds with dimensions of 40 mm^3^ × 40 mm^3^ × 5 mm^3^. The valve is positioned between them and is in contact with the plates. The valve geometry is described in detail earlier in Section 2.3. The Mooney–Rivlin equations presented in the previous section are used to describe the mechanical behavior of the valve. The finite element mesh consisted of tetrahedrons described by a quadratic function. The mesh element size at the valve leaflet tips is 0.5 mm, while on the remaining parts it is 1 mm. The valve position was identical to that in the experiment. The lower plate was fixed, while the upper plate moved vertically downward at a speed of 30 mm/min.

**FIGURE 5:**
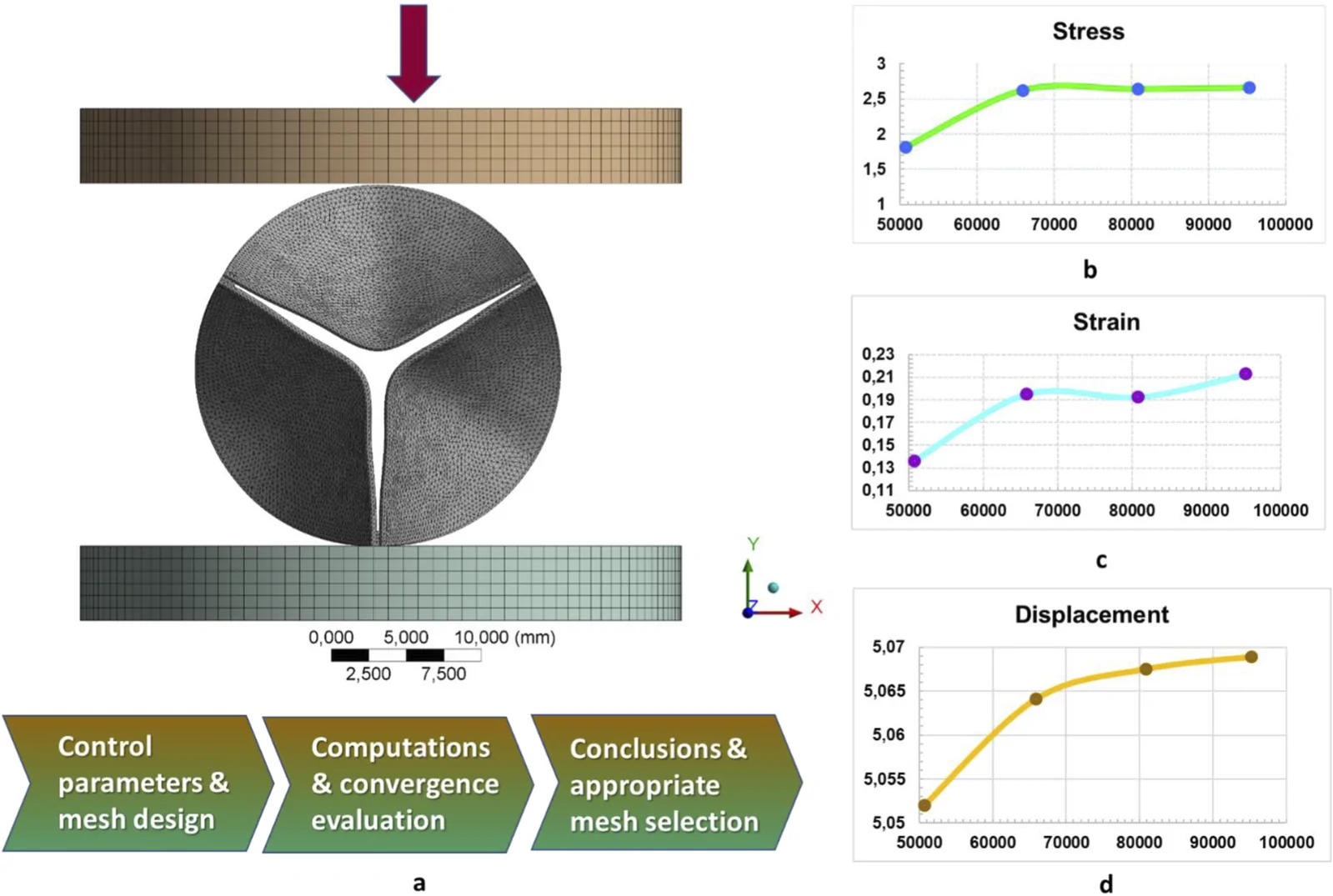
Finite element mesh and convergence study: **(a)** – numerical model and finite element mesh, **(b)** – mesh independency test result (stress distribution, MPa), **(c)** – mesh independency test result (strain distribution), **(d)** – mesh independency test result (displacement distribution, mm).

A contact type “Bonded” was set between the plates and the lateral surface of the valve. The contact for the leaflet–leaflet and leaflet–plate surfaces was specified as friction (frictional contact), with a friction coefficient of 0.2. The computation stopped when the displacement of the upper plate reached 10 mm. Mathematical formulation is presented in Supplementary Material 2.

The convergence analysis enabled choosing the optimal mesh element size. Four meshes were analyzed (mesh 1 consisted of 50,706 elements, mesh 2 consisted of 65,902 elements, mesh 3 consisted of 80,840 elements, mesh 4 consisted of 95,278 elements). While all the meshes led to the converged solution since the difference in results of the computations (stress, strain and displacement) were negligible in the case of mesh 2 and mesh 3 (less than 2%). A mesh convergence study was performed to ensure that the uncertainty associated with spatial discretization is not significant. It took more time to simulate the latter mesh (mesh 4), but the differences in the results were negligible, thus it can be concluded that results are independent of the mesh. Comparison between solid meshes is presented in Figures 5b–d. Finally, mesh 3 was chosen for subsequent analysis. Thus, solid meshes consisted of 80,840 elements and 218,078 nodes was used for the final computations.

## 3 Results

### 3.1 TPU and SEBS tensile tests with 50, 100 и 500 mm/min loading rates

The results of the tensile experiment on dogbone specimens made of TPU and SEBS at speeds of 50, 100, and 500 mm/min are shown in Figure 6. The TPU tensile specimen exhibits the highest stress/strain values at a speed of 100 mm/min, regardless of the filament alignment pattern. Slightly lower results are observed at a speed of 50 mm/min, which is likely associated with a wide range of plastic deformation. In the case of SEBS, a different pattern is observed: at 500 mm/min, it demonstrates high resistance to fracture, unlike TPU. A comparison of TPU and SEBS specimens at the same speeds and with the same alignment, based on maximum stress values, shows a minimal difference of 4% with the axial alignment at a speed of 50 mm/min; however, when comparing strains, the difference increases to 90%. This is due to TPU exhibiting greater strength and ductility compared to SEBS.

**FIGURE 6:**
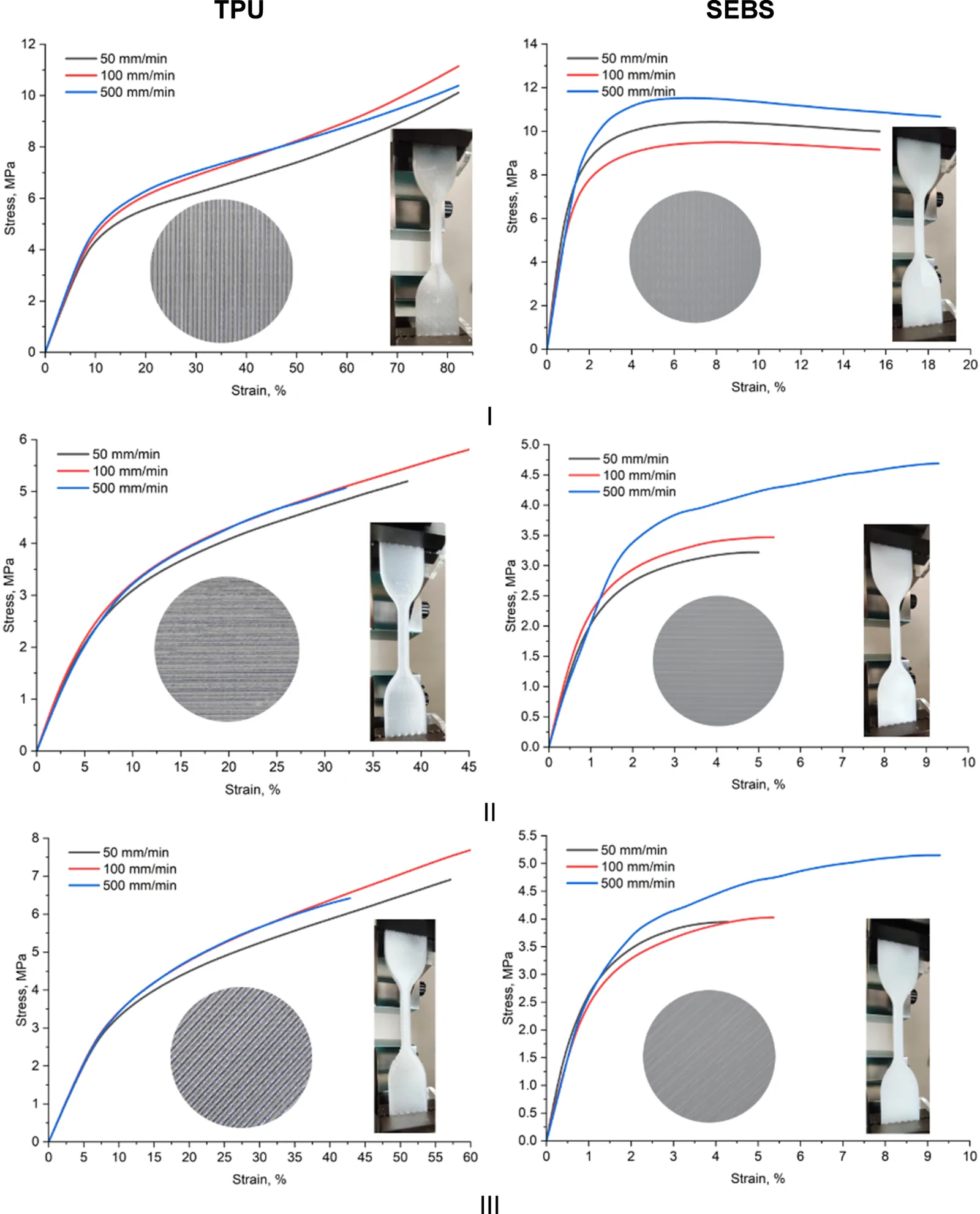
Stress–strain curves of TPU and SEBS tensile test at loading rates of 50, 100, 500 mm/min for various filament alignments: **(I)** – axial; **(II)** – transversal; **(III)** – diagonal.

### 3.2 TPU and SEBS tensile tests with 100 mm/min loading rate

Figure 7 presents the results of the tensile experiment on dog-bone specimens made of TPU and SEBS at a speed of 100 mm/min. In the case of tensile testing with axial alignment of TPU or SEBS specimens, a very close correspondence in mechanical behavior between the specimens is observed. The samples that did not break were excluded from the calculation of maximum strain and fracture strain. The stress-strain curves of SEBS specimens, regardless of filament alignment, are more concave upward than those of TPU but exhibit lower strain values. When pairwise comparing stress and strain values between the alignments of TPU and SEBS, a consistent trend can be observed: TPU specimens always exhibit the highest values.

**FIGURE 7:**
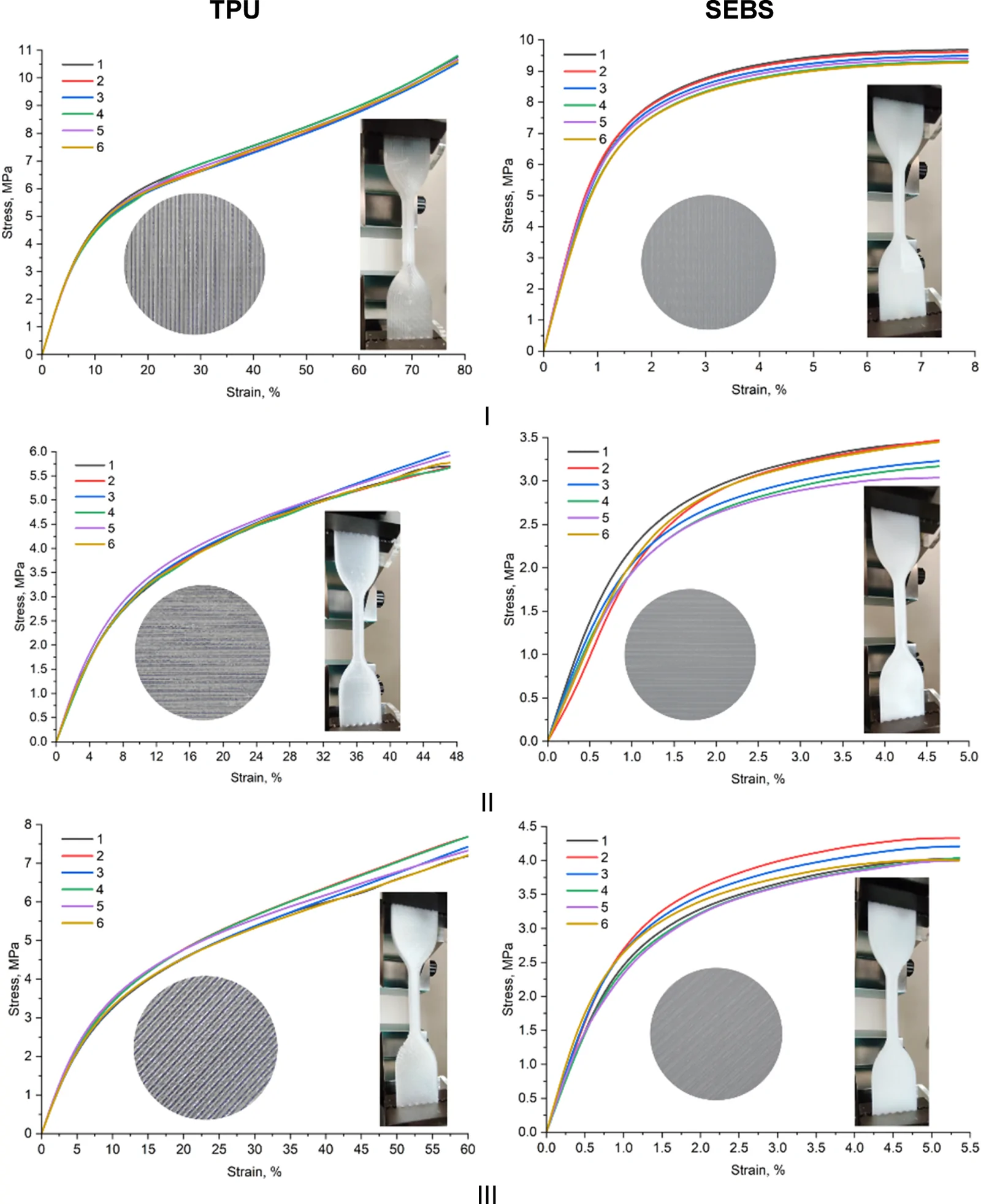
Stress–strain curves of TPU and SEBS tensile test at loading rate of 100 mm/min for various filament alignments: **(I)** – axial; **(II)** – transversal; **(III)** – diagonal.

During the experiment, Young’s modulus was determined for both materials with different filament alignment patterns. The results obtained are presented in Table 7. The SEBS material exhibits higher Young’s modulus values compared to TPU in all alignment configurations. The average Young’s modulus for SEBS exceeds that of TPU by more than 5 times. For both TPU and SEBS, the maximum Young’s modulus values are observed with the axial orientation, while the minimum values occur with the transversal orientation. Tables 7, 8 contains Young’s modulus and Poisson’s ratio values for TPU and SEBS samples.

**TABLE 7:** Young’s moduli of TPU and SEBS specimens with various filament alignment from tensile tests.

Filament alignment	TPU Young’s modulus, MPa	SEBS Young’s modulus, MPa
Axial	28.49 ± 2.83	174.71 ± 17.06
Transversal	22.50 ± 2.15	97.34 ± 9.42
Diagonal	24.04 ± 2.31	105.15 ± 10.33

**TABLE 8:** Poisson’s ratios of TPU and SEBS specimens with various filament alignment from tensile tests.

Filament alignment	TPU Poisson’s ratio	SEBS Poisson’s ratio
Axial	0.45 ± 0.042	0.36 ± 0.035
Transversal	0.12 ± 0.015	0.14 ± 0.018
Diagonal	0.24 ± 0.028	0.21 ± 0.026

### 3.3 TPU and SEBS DIC tensile tests results

The evolution of the maximum principal strain in SEBS specimens with axial (0°) filament alignment is presented in Figure 8. At the initial loading stage (t_0_ = 5 s), the strain field is distributed relatively uniformly over the gauge section, with the highest values located near the specimen center. As tensile deformation progresses, strain localization gradually develops within the gauge section. By t_1_ = 10 s, the maximum principal strain in the emerging necking region reaches approximately 0.15–0.20. Immediately before failure (t_2_ = 35 s), deformation becomes highly localized within a narrow band, where the maximum principal strain exceeds 0.35. At this stage, a pronounced strain gradient extends from the necking region toward the gripping ends, reflecting the progressive concentration of deformation prior to fracture.

**FIGURE 8:**
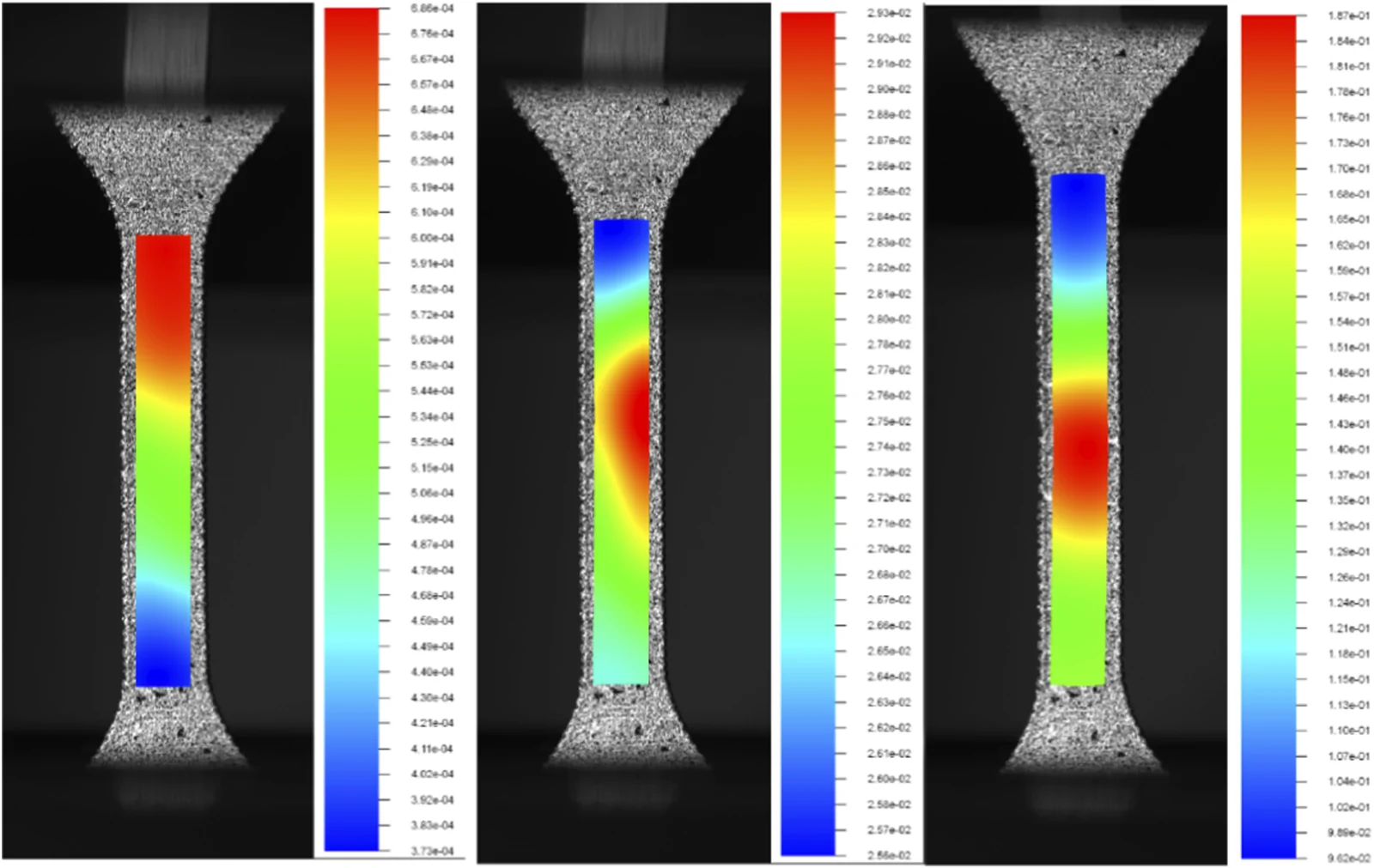
Evolution of principal strain of SEBS sample (axial filament alignment) ( $t_{0}=5\;s,t_{1}=10\;s,t_{2}=35\;s$ ).

Figure 9 presents the evolution of the maximum principal strain in SEBS specimens with diagonal (45°) filament alignment during tensile loading. At the initial stage, the strain field is distributed more uniformly across the gauge section than in specimens with axial filament alignment. By t_1_ = 10 s, localized deformation begins to develop, with maximum principal strain values of approximately 0.12–0.15. Compared with the axial configuration, the localized region remains broader and less pronounced. Immediately before failure (t_2_ = 35 s), strain localization extends over a wider area of the gauge section rather than forming a narrow necking band. This deformation pattern is consistent with the higher Young’s modulus measured for the diagonal orientation (105.15 MPa) relative to the axial configuration (97.34 MPa).

**FIGURE 9:**
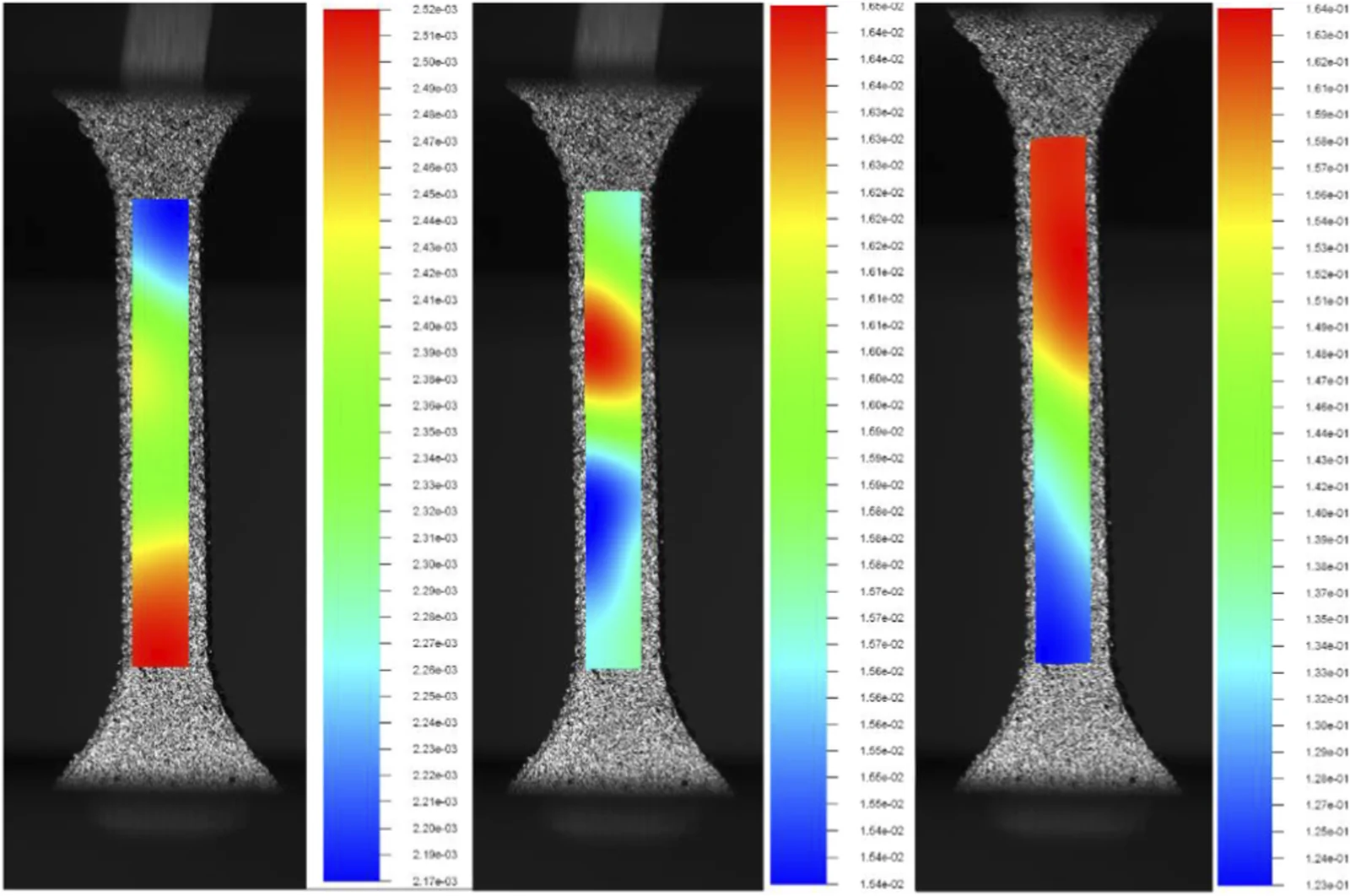
Evolution of principal strain of SEBS sample (diagonal filament alignment) ( $t_{0}=5\;s,t_{1}=10\;s,t_{2}=35\;s$ ).

Figure 10 presents the evolution of the maximum principal strain in SEBS specimens with transversal (90°) filament alignment. Among the three filament orientations, the transversal configuration exhibits the most heterogeneous strain distribution. At the initial loading stage (t_0_ = 5 s), several localized strain regions are already visible along the gauge section. After 10 s of loading, the maximum principal strain reaches approximately 0.08–0.10, remaining lower than the corresponding values observed for the axial and diagonal orientations. Immediately before failure (t_2_ = 35 s), deformation is primarily localized near the specimen edges, where the maximum principal strain approaches 0.25.

**FIGURE 10:**
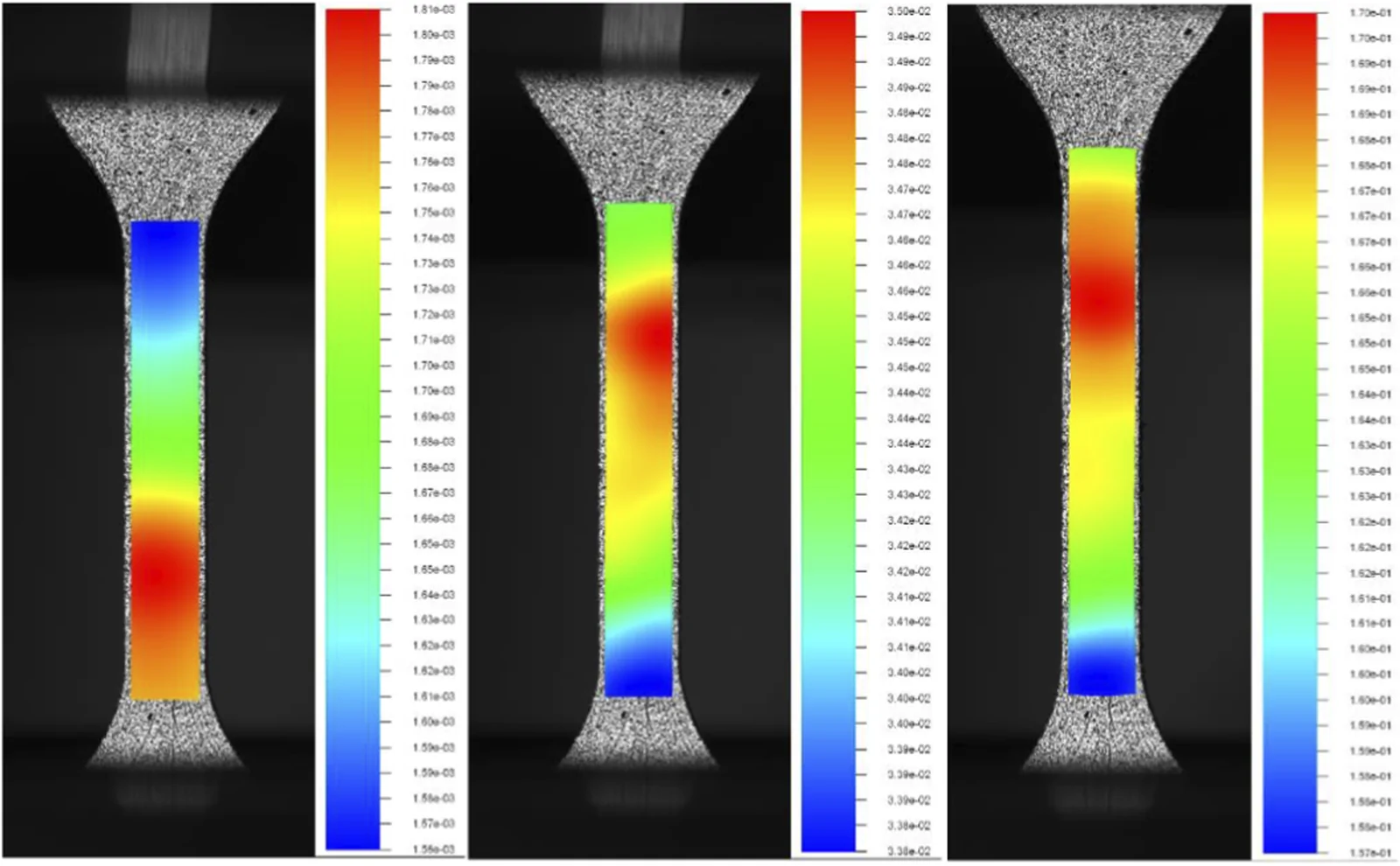
Evolution of principal strain of SEBS sample (transversal filament alignment) ( $t_{0}=5\;s,t_{1}=10\;s,t_{2}=35\;s$ ).

The evolution of the maximum principal strain in TPU specimens with axial (0°) filament alignment is shown in Figure 11. Failure occurs at approximately 60 s, whereas SEBS specimens tested under the same conditions fail after about 35 s. At t_0_ = 5 s, the strain field is distributed relatively uniformly across the gauge section. As loading progresses, localized deformation develops in the central region, with maximum principal strain values of approximately 0.10–0.12 at t_1_ = 10 s. Immediately before failure (t_2_ = 60 s), a distinct necking zone forms within the gauge section, where the maximum principal strain exceeds 0.60. Compared with SEBS, TPU develops substantially higher strain before fracture (approximately 0.60 versus 0.35), reflecting its greater deformability under tensile loading.

**FIGURE 11:**
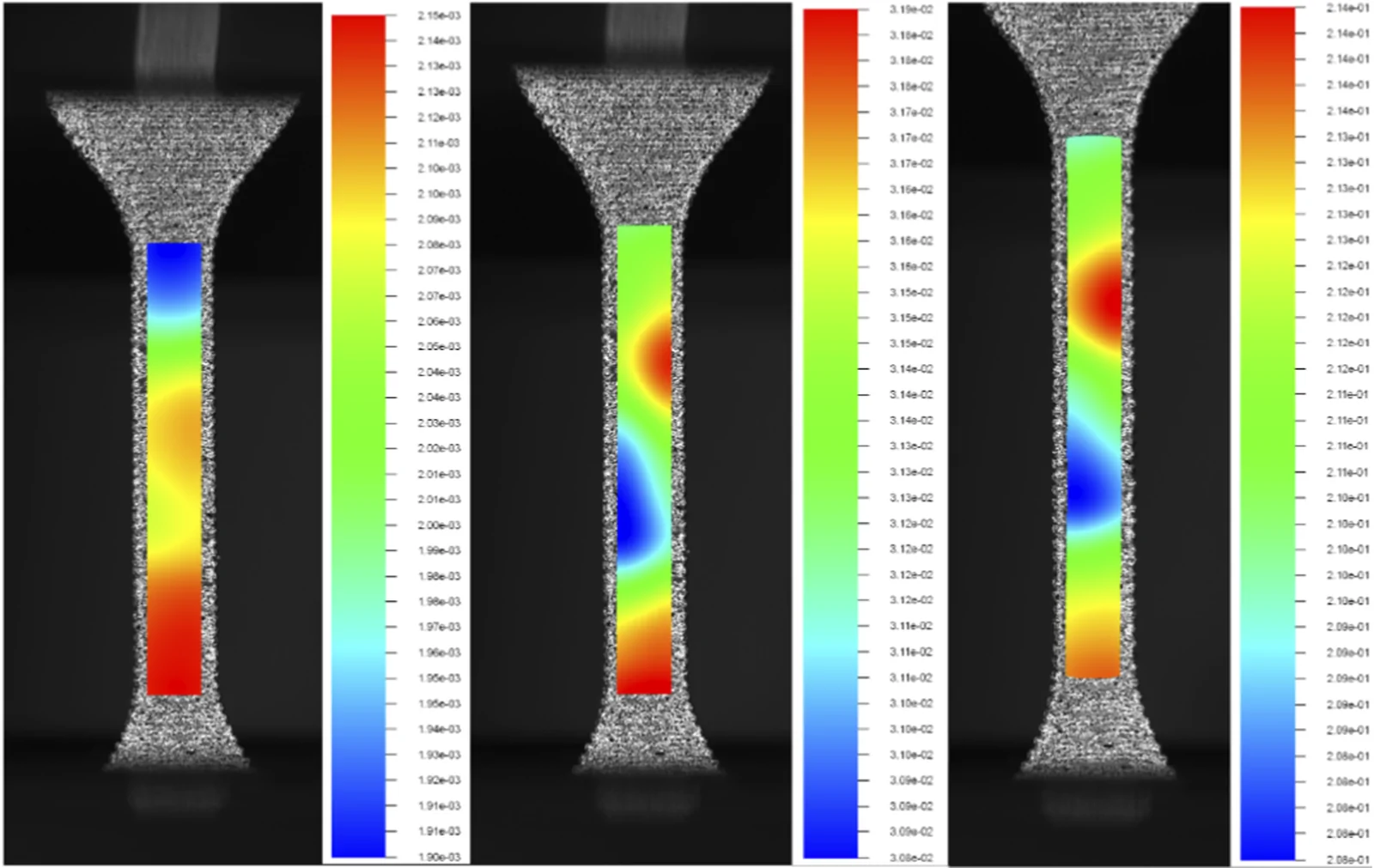
Evolution of principal strain of TPU sample (axial filament alignment) ( $t_{0}=5\;s,t_{1}=10\;s,t_{2}=60\;s$ ).

The evolution of the maximum principal strain in TPU specimens with diagonal (45°) filament alignment under tensile stress is shown in Figure 12. The strain field is distributed fairly evenly throughout the gauge section at the start of the test (t_0_ = 5 s), with only slight concentrations close to the specimen edges. The maximal primary strain reaches roughly 0.08–0.10 after 10 s of loading. Deformation concentrates in the center region just prior to failure (t_2_ = 60 s), where the greatest primary strain is close to 0.55. A more progressive distribution of deformation across the gauge section is shown by the localized region’s larger and less clearly defined shape when compared to the axial design.

**FIGURE 12:**
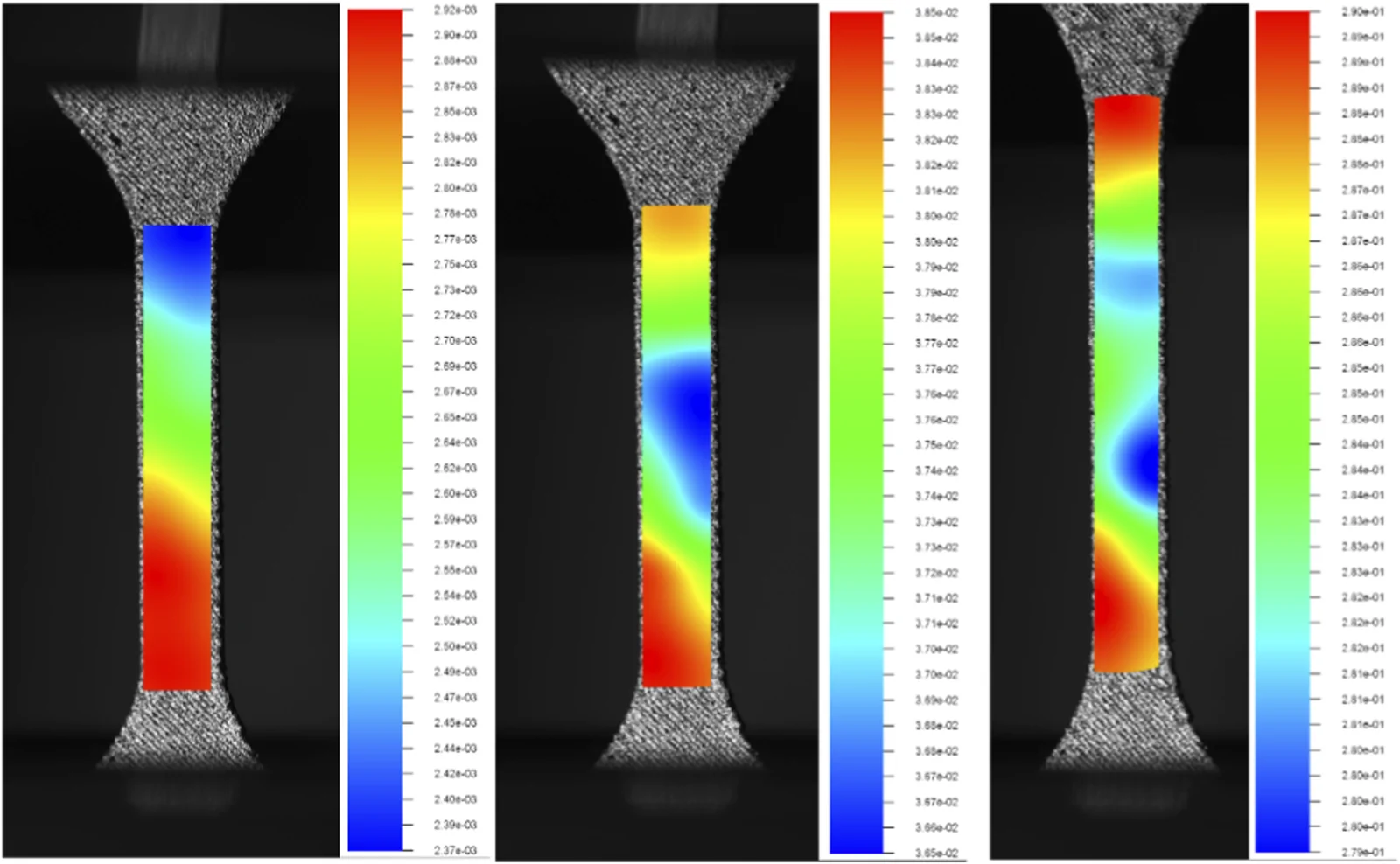
Evolution of principal strain of TPU sample (diagonal filament alignment) ( $t_{0}=5\;s,t_{1}=10\;s,t_{2}=60\;s$ ).

The strain evolution in TPU specimens with transverse (90°) filament alignment is shown in Figure 13. Localized strain patches are already apparent from the beginning of loading, in contrast to the other filament orientations. The highest primary strain is roughly 0.06–0.08 at t_1_ = 10 s. The gauge section develops many localization bands as loading increases, and the maximum principal strain reaches around 0.50 just prior to failure (t_2_ = 60 s). Compared to the axial and diagonal arrangements, the resulting strain field exhibits greater heterogeneity.

**FIGURE 13:**
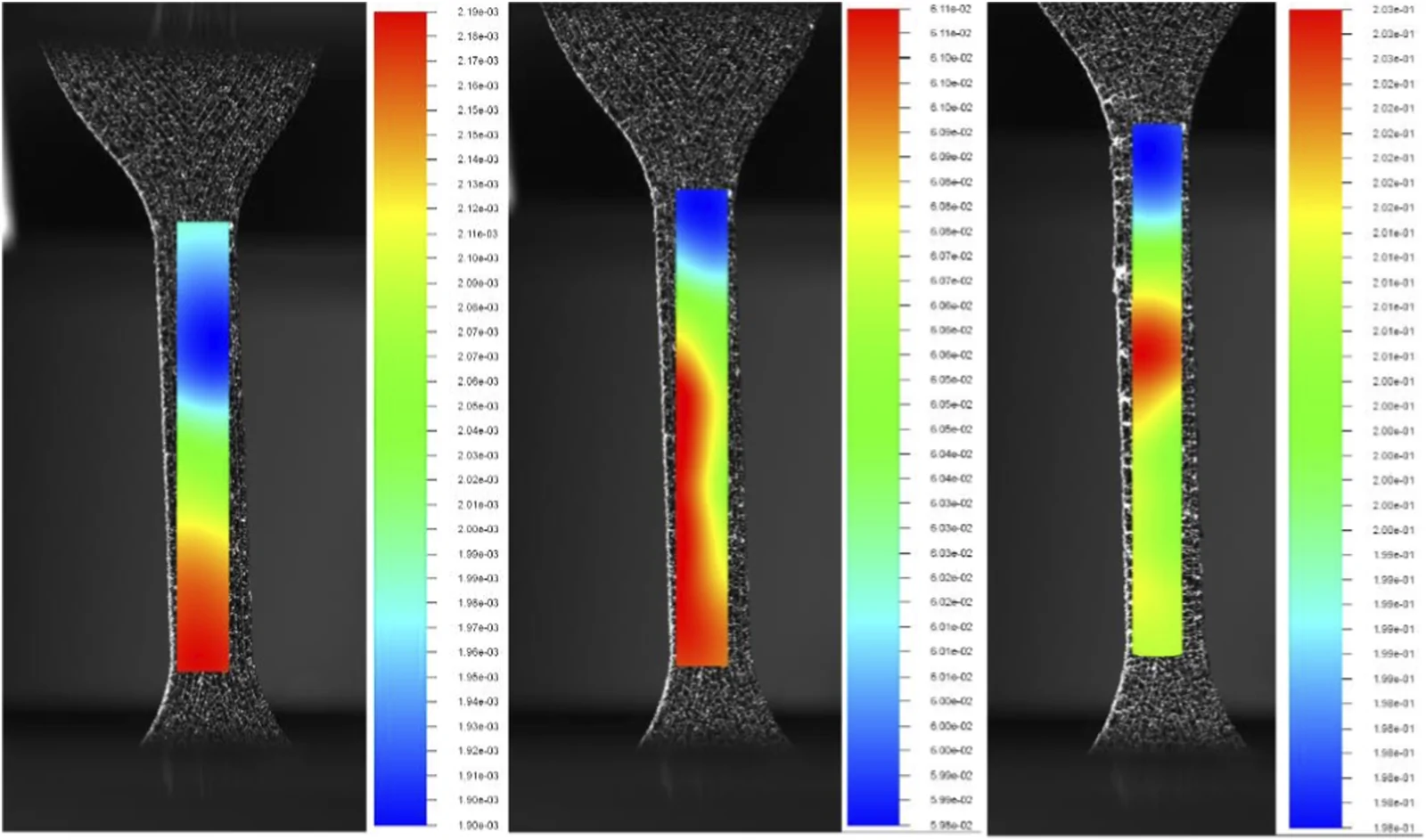
Evolution of principal strain of TPU sample (transversal filament alignment) ( $t_{0}=5\;s,t_{1}=10\;s,t_{2}=60\;s$ ).

For all filament orientations, the DIC measurements consistently reveal greater maximum principal strains in TPU than in SEBS. The variation in maximum principal strain varies between roughly 40% and 70%, depending on the filament configuration. While the transversal orientation results in a more dispersed strain field, axial alignment produces the strongest localization of deformation for both materials. These findings unequivocally show that filament orientation affects the amount and spatial distribution of strains during tensile deformation. This is a crucial factor to take into account when designing 3D-printed aortic valve leaflets.

### 3.4 TPU and SEBS compression tests with 1 mm/min loading rate

The results of the compression experiment on TPU and SEBS cubes at a speed of 1 mm/min are shown in Figure 14. On the stress-strain graphs for SEBS, regardless of the alignment, a rounding (plateau) is observed at the end of the curves; such mechanical behavior is likely associated with the stiffness of SEBS. Comparison of the maximum stress values between the alignments of TPU and SEBS consistently shows a difference of 70%, although the strain magnitude is the same, indicating a weak influence of the specimen alignment type on the compressive strength of the material. During the experiment, Young’s modulus was determined for both materials with different alignment patterns. The results obtained are presented in Table 9. A comparative analysis between TPU and SEBS shows that SEBS exhibits higher Young’s modulus values in all alignment types. The difference between the computed Young’s modulus values of the materials averages more than 3 times. Maximum stiffness of the materials is achieved with the axial alignment, while minimum stiffness occurs with the transversal alignment. The results in Table 9 show that for TPU the choice of alignment type is less critical, as the spread of Young’s modulus values is small, whereas for SEBS the filament alignment has a significant effect.

**FIGURE 14:**
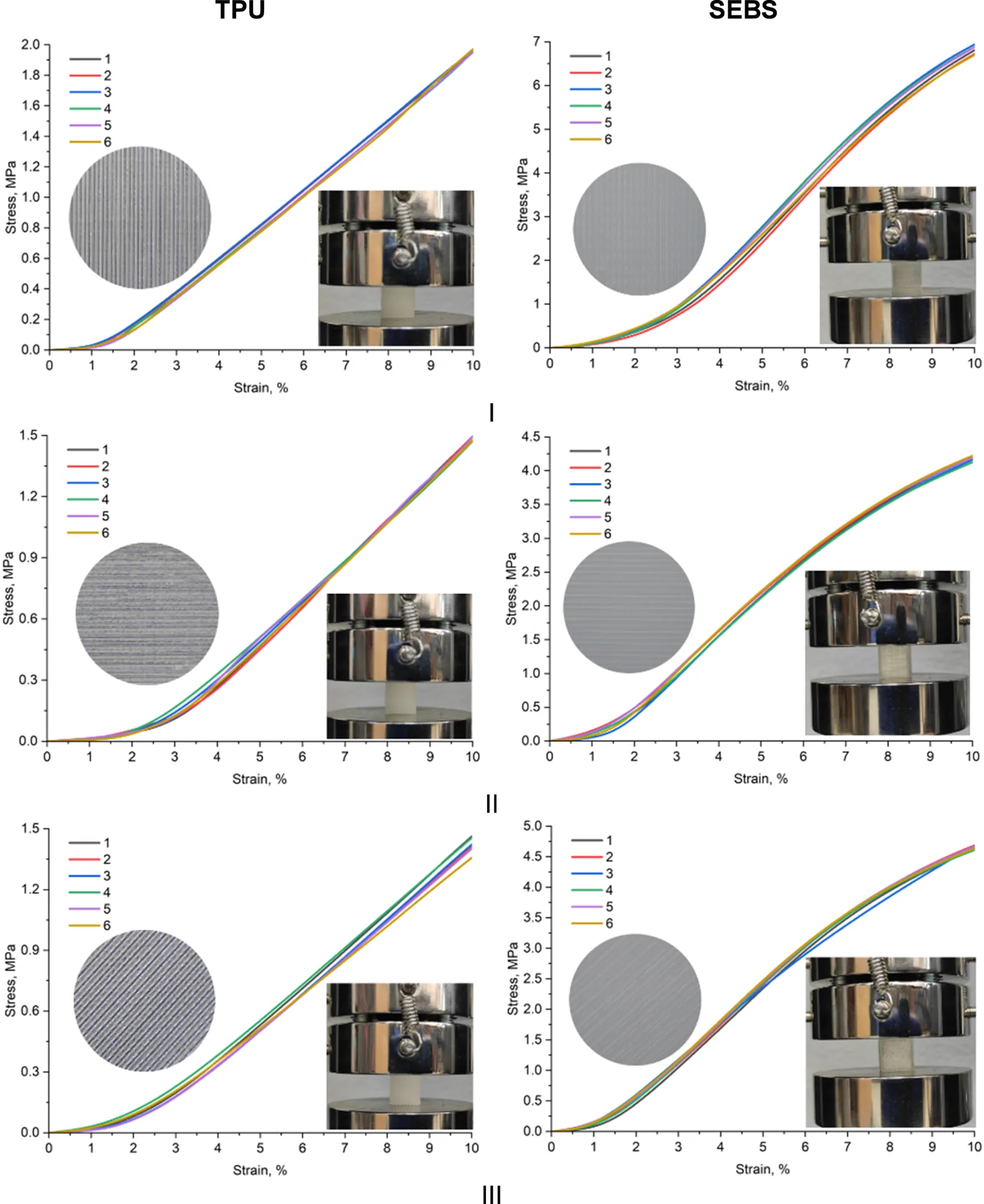
Stress–strain curves of TPU and SEBS compression test at loading rate of 1 mm/min: **(I)** – axial filament alignment; **(II)** – transversal filament alignment; **(III)** – diagonal filament alignment.

**TABLE 9:** Young’s moduli of TPU and SEBS specimens with various filament alignment from compression tests, MPa.

Filament alignment	TPU	SEBS
Axial	16.62 ± 1.59	60.08 ± 5.72
Transversal	11.70 ± 1.14	39.51 ± 3.85
Diagonal	12.11 ± 1.1	43.87 ± 4.21

### 3.5 TPU and SEBS compression tests with 30 mm/min loading rate

The results of the compression experiment on aortic valve specimens made of TPU and SEBS at a speed of 30 mm/min are shown in Figure 15. The SEBS aortic valve specimen is stiffer than the TPU one, which is clearly evident from the slope of the displacement–force curves. TPU demonstrates a greater ability to compress with less resistance at the initial stage, whereas SEBS, on the contrary, begins to resist compression earlier and more strongly, indicating lower compliance. The spread of final force values for TPU is considerably lower, amounting to 0.09 N (4% of the maximum case), while for SEBS the spread increases to 2.66 N (20% of the maximum case).

**FIGURE 15:**
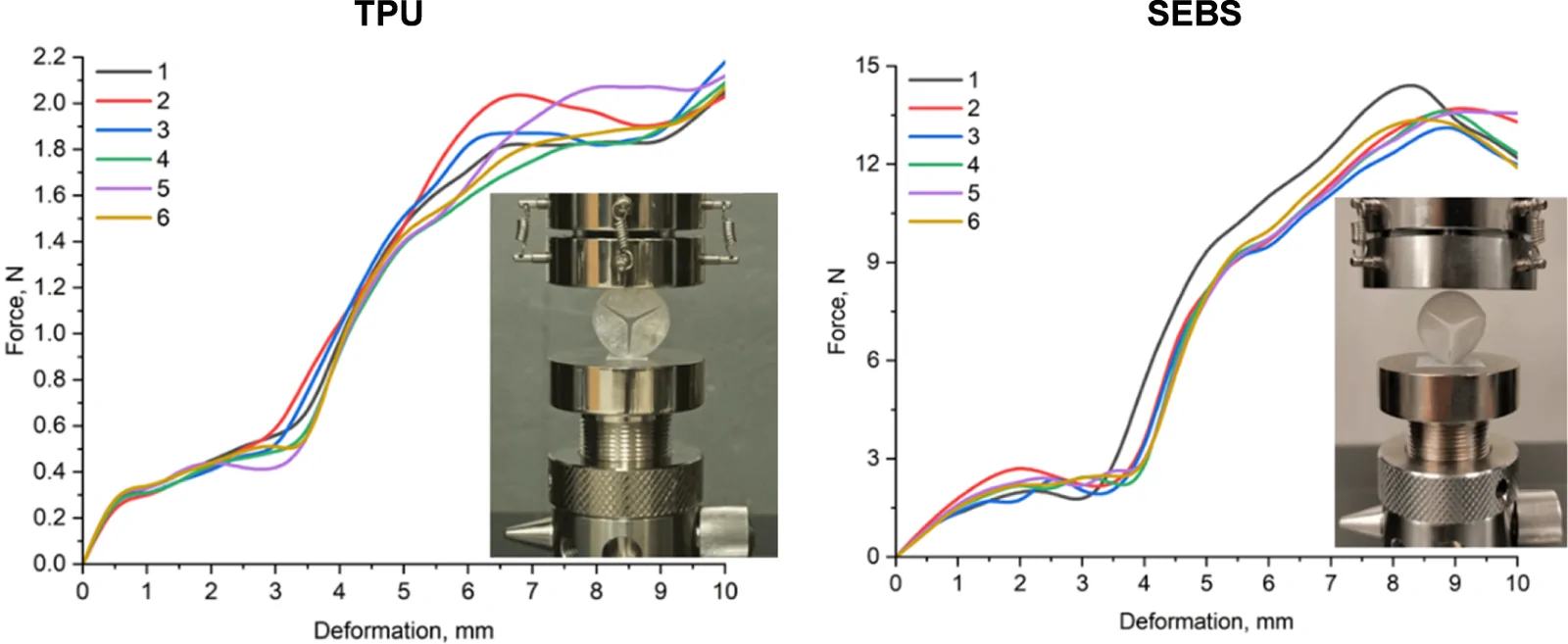
Curves force–displacement for TPU and SEBS aortic valve leaflets compression.

### 3.6 Numerical and DIC aortic valve compression tests results

Figure 16 compares the vertical displacement fields of the TPU aortic valve between the experiment (DIC) and the numerical model at various time points. The vertical displacement fields of the DIC aortic valve at 50 s reach a maximum value of 4.2 mm and differ from those of the numerical model of the TPU valve by 5%. Visually, Figure 16 shows the upper leaflet of the aortic valve bending upward, the lateral leaflets converging, and the gap between them disappearing, while the valve base takes on an elliptical shape.

**FIGURE 16:**
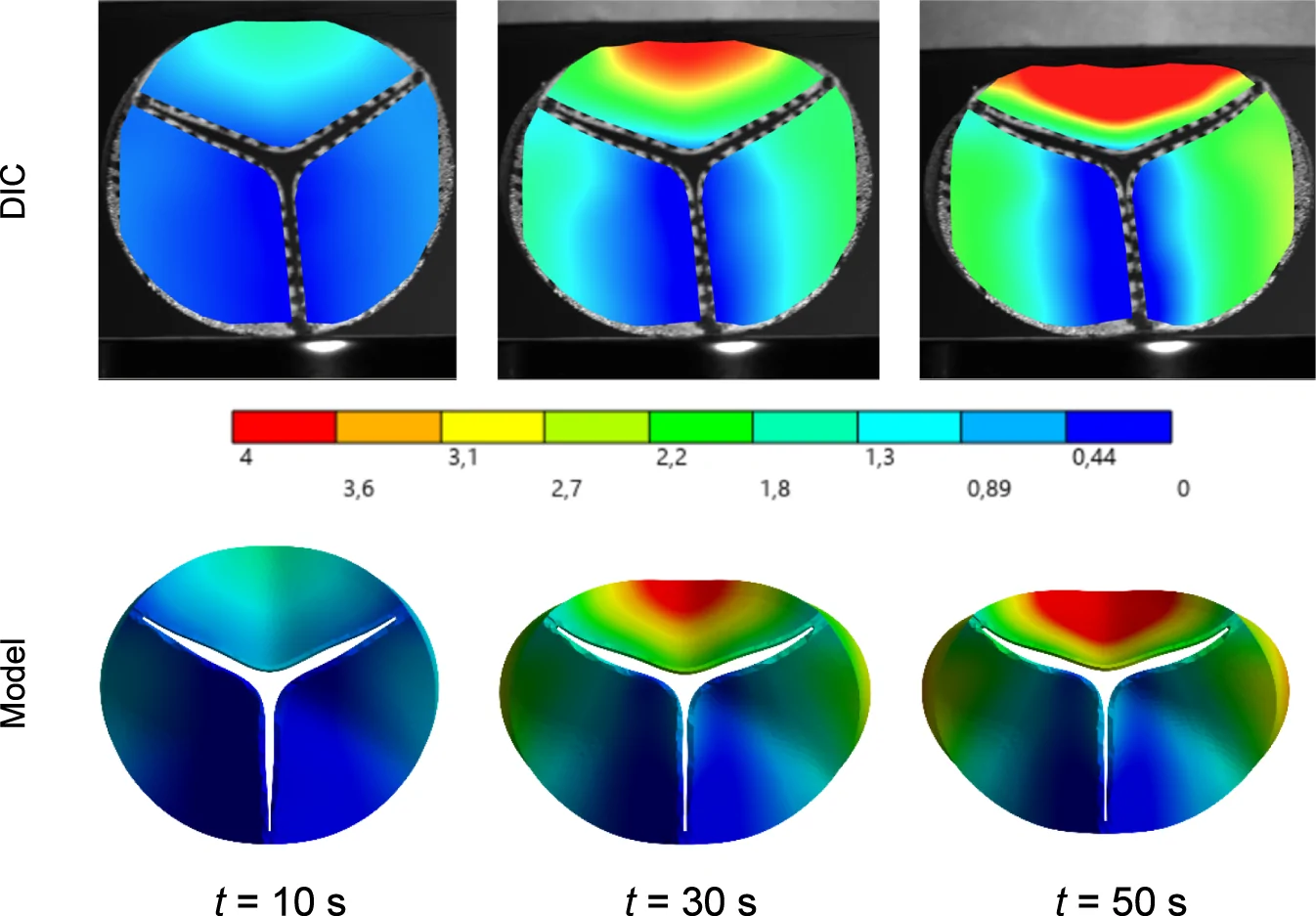
Numerical and DIC displacement distributions of TPU aortic valve leaflets compression at various time, mm.

Figure 17 compares the vertical displacement fields of the SEBS aortic valve between the experiment (DIC) and the digital model at various time points. The vertical displacement fields of the DIC aortic valve at 50 s reach a maximum value of 4.1 mm and differ from the digital model of the TPU valve by 10%. Visually, Figure 17 shows the upper leaflet of the aortic valve bending upward; the lateral leaflets do not converge, and the gap between them has not changed; furthermore, small displacements are observed at the commissures of the leaflets.

**FIGURE 17:**
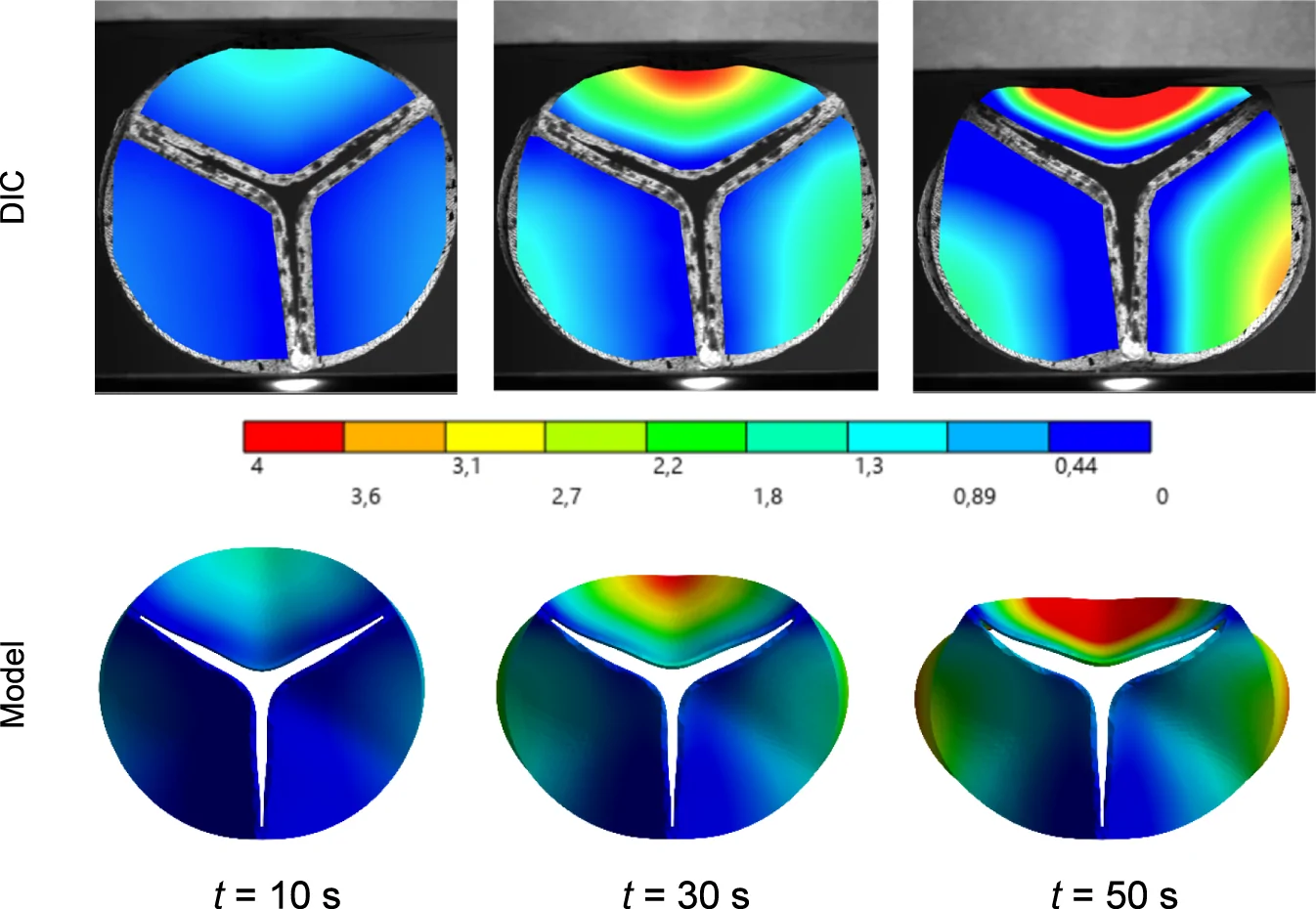
Numerical and DIC displacement distributions of SEBS aortic valve leaflets compression at various time, mm.

Using the digital image correlation method, the vertical displacement fields were monitored during the contraction of the aortic valve leaflets made of TPU and SEBS, which were 3D-printed. The vertical displacement fields during closure at a speed of 30 mm/min are shown in Figures 16, 17 at three time points: 10 s, 30 s, and 50 s.

At time t = 10 s, after contact with the upper plate, displacements begin to appear in the upper parts of the valve flaps. While the valve base remains stable, the displacements in the upper parts increase to approximately 0.89–1.3 mm. As one might expect, the displacement gradually decreases from top to bottom. Deformation has already begun, as evidenced by the slight inward displacement of the lateral leaflets. The behavior described above is identical for both TPU and SEBS materials.

The difference between the vertical displacement fields of the experimental (DIC) TPU and SEBS aortic valves in Figures 16, 17 is evident in the two upper commissures of the leaflets relative to each other at time points of 30 s and 50 s, respectively. Consequently, the SEBS material exhibits greater internal resistance to compression, which accounts for the greater stiffness of this valve.

## 4 Discussion

The present research in the field of polymeric heart valves (PHVs) is attracting much attention and scientists are more and more focused on the development of new biomaterials that can overcome the limitations of traditional bioprosthetic and mechanical valves. PHVs have the potential to combine the mechanical durability of synthetic materials with the biocompatibility and hemodynamic functionality of natural valves, potentially overcoming problems like the limited durability of tissue-based bioprostheses and the lifelong anticoagulation therapy needed for mechanical valve replacements. One promising direction in this area is the use of fused deposition modeling (FDM) 3D printing technologies that can be used to produce complex and patient-specific valve designs with tailor-made mechanical properties.

### 4.1 Influence of filament alignment and printing parameters on mechanical properties

The results of the tensile tests revealed a pronounced anisotropic behavior for both TPU and SEBS specimens, which is consistent with the inherent characteristics of the FDM process. Thermoplastic polyurethane (TPU) is typically modeled as an isotropic hyperelastic material; however, when fabricated using FDM, it exhibits anisotropic behavior due to directional filament alignment (Martín-Sosa et al., 2025; Raf et al., 2026). The present study demonstrated that specimens with axial filament alignment achieved the highest tensile strength and Young’s modulus for both materials, while transversal (90°) alignment consistently yielded the lowest mechanical performance. This finding correlates with data on filament alignment effect on the mechanical properties of 3D-printed parts, with 0° raster aligned with the loading direction demonstrating the highest tensile strength and modulus, while 90° raster displayed the lowest performance due to weak interlayer bonding (Fuentes del Toro et al., 2025; Wilińska et al., 2025; Raf et al., 2026).

The maximum stress values for TPU were higher at a loading rate of 100 mm/min than at 50 and 500 mm/min, indicating a complicated rate-dependent response (Reyes et al., 2024). This finding is in line with TPU’s hyperelastic and viscoelastic characteristics, which call for complex constitutive frameworks that may represent significant deformations and rate-dependent reactions under pulsatile flow circumstances (Bruère et al., 2023). SEBS, on the other hand, showed the greatest resistance to fracture at 500 mm/min, suggesting that this copolymer responds differentially to different strain rates (Fan et al., 2015). Under physiological conditions, where leaflets experience cyclic loading at frequencies corresponding to heart rhythms, these variations in rate sensitivity may have significant effects on valve performance (Stanová et al., 2024; Pil et al., 2026a).

The influence of layer height and extrusion temperature on interlayer bonding should also be acknowledged. Previous studies have shown that reducing layer height can improve interlayer bonding but may narrow raster-to-raster bond widths (Tarrés et al., 2025). The printing parameters were optimized through preliminary trials to achieve sufficient interlayer cohesion for both materials (Maroszek et al., 2025). However, the significant difference in Young’s modulus between TPU and SEBS—with SEBS exceeding TPU by more than five times in tension—suggests that the molecular structure and thermal history during printing play a crucial role in determining the final mechanical properties (Liu et al., 2023; Lang et al., 2025; Daly et al., 2026).

### 4.2 Comparison of TPU and SEBS mechanical performance

TPU was shown to be more ductile than SEBS, with elongation values for TPU specimens at similar loading circumstances being about 90% higher (Quiñonez et al., 2021). The two polymers’ different molecular architectures are responsible for this difference in mechanical behavior: TPU, a thermoplastic polyurethane, has a segmented structure with hard and soft segments that allow for extensive molecular rearrangement under load (Jin et al., 2020), whereas SEBS, a styrenic block copolymer, has a more rigid polystyrene endblock domain structure that restricts extensibility (Dechnarong et al., 2021). Applications needing more leaflet rigidity may benefit from SEBS’s increased stiffness, which is demonstrated by its noticeably higher Young’s modulus (97.34–174.71 MPa under tension depending on alignment) (Todesco et al., 2025). However, the lower ductility of SEBS (elongation at break ∼400–710%) compared to TPU could potentially limit its fatigue resistance under the cyclic loading conditions experienced by aortic valve leaflets (Wang et al., 2023).

SEBS is notably stiffer than TPU, as demonstrated by compression tests on cuboid specimens, where Young’s modulus values are more than three times higher than those of TPU in all filament alignments. SEBS leaflets showed noticeably greater resistance to compression in the compression tests of the 3D-printed aortic valve leaflets, as shown by the steeper slope of the force-displacement curves. The mechanical response of SEBS leaflets was more variable, as seen by the spread of final force values being wider for SEBS (20%) than for TPU (4%).

### 4.3 Implications for TAVI device design

The design of polymeric TAVI devices is directly impacted by the noted mechanical property variations between TPU and SEBS. The particular clinical requirements and the intended leaflet performance characteristics should direct the selection of these materials (Mao P. et al., 2025). Because of its higher ductility and reduced stiffness, TPU may be the material of choice for applications needing more compliant leaflets with less resistance to opening and more capacity to adjust to the aortic root architecture. On the other hand, despite its reduced ductility, SEBS may be advantageous in applications where increased leaflet rigidity is required.

The mechanical behavior of these materials was further clarified by the numerical simulation of leaflet compression. Compared to TPU panels, the maximum equivalent stress in SEBS panels was twenty times higher, at 82.8 MPa. The large difference in Young’s modulus between the two materials is directly responsible for this substantial differential in internal stress generation. In SEBS specimens, the localization of stresses at the leaflet attachment locations suggests possible failure risk areas under cyclic loading, which calls for additional research using fatigue testing. SEBS-based PHVs have shown little cytotoxicity, hemolysis, and adverse immunological reactions in preclinical experiments, and prior research has shown that SEBS provides exceptional thermal stability, flexibility, and fatigue resistance. However, the stress concentrations found in this study indicate that design modification may be required to reduce the likelihood of fatigue-induced failure at attachment locations, possibly by adding structural reinforcements or graded material characteristics (Li et al., 2019).

The anisotropic nature of both TPU and SEBS, arising from the FDM process, also has important implications for valve design. The layer-by-layer deposition introduces direction-dependent mechanical properties that are not present in bulk materials. This anisotropy must be accounted for in both the design and numerical simulation of PHVs. The development of accurate constitutive models that capture the hyperelastic and anisotropic behavior of 3D-printed TPU and SEBS is therefore essential for reliable fluid–structure interaction analysis and virtual testing of novel TAVI devices (Evangelista et al., 2025).

### 4.4 Comparison with native tissue and existing materials

The mechanical properties of TPU and SEBS leaflets should be contextualized relative to native human aortic valve tissue and existing materials used in bioprosthetic valves. Native aortic valve leaflets exhibit anisotropic, nonlinear mechanical behavior with a complex layered structure (Stella and Sacks, 2007). The elastic modulus of native leaflets ranges from approximately 10–30 MPa depending on the direction of loading and the specific layer (Koch et al., 2010). In this context, the Young’s modulus values obtained for TPU (11.70–16.62 MPa in compression) fall well within the range of native tissue, suggesting that TPU leaflets could potentially mimic the mechanical behavior of the native valve more closely (Hasan et al., 2014). In contrast, SEBS leaflets (39.51–60.08 MPa in compression) are substantially stiffer than native tissue, which may affect both the opening and closing dynamics of the valve.

Compared to glutaraldehyde-fixed bovine pericardium (elastic modulus ∼100–300 MPa), both TPU and SEBS are more compliant, which may translate to improved hemodynamic performance through larger effective orifice areas and lower transvalvular pressure gradients (Zioupos et al., 1994). However, the lower stiffness of these polymers also raises concerns about leaflet prolapse and coaptation under physiological pressures, particularly for SEBS which, despite being stiffer than TPU, still falls below the stiffness of fixed pericardium (Sugimoto and Sacks, 2013). These considerations highlight the need for careful design optimization, potentially incorporating leaflet thickness variations or structural reinforcements to achieve the desired balance of compliance and coaptation.

### 4.5 Study limitations and future directions

Several limitations of the present study should be acknowledged. First, the tensile and compression tests were conducted under quasi-static loading conditions at room temperature, whereas aortic valve leaflets experience complex, dynamic loading under physiological conditions (37 °C, pulsatile flow). The rate-dependent behavior observed for both materials underscore the importance of conducting mechanical characterization under conditions that more closely approximate the *in vivo* environment. Future studies should incorporate dynamic mechanical analysis and fatigue testing under physiologically relevant cyclic loading to better assess the long-term durability of 3D-printed TPU and SEBS leaflets.

Second, the present study focused on the mechanical characterization of FDM-printed specimens without considering the potential effects of sterilization, which is a critical step in the manufacturing of medical devices. The impact of sterilization methods (e.g., ethylene oxide, gamma irradiation, or autoclaving) on the mechanical properties and biocompatibility of TPU and SEBS requires systematic investigation.

Third, the development of more sophisticated constitutive models that capture the viscoelastic and anisotropic behavior of 3D-printed TPU and SEBS is necessary for accurate fluid–structure interaction analysis and the prediction of valve performance under physiological conditions. It was demonstrated that Mooney–Rivlin models produce good approximation for the whole stress–strain curve of TPU and SEBS, and future work should extend these models to capture the anisotropic effects observed in this study.

Fourth, the long-term biocompatibility and hemocompatibility of FDM-printed TPU and SEBS leaflets require further investigation. While SEBS has demonstrated excellent biocompatibility in previous studies, the surface characteristics of FDM-printed parts—including roughness and the presence of residual processing aids—may affect platelet adhesion and thrombogenicity. Surface modification strategies may be necessary to enhance the hemocompatibility of these materials for blood-contacting applications.

While TPU and SEBS show promise as materials for further investigation, broader conclusions regarding their suitability for clinical TAVI applications—including hydrodynamic performance, fatigue resistance under cyclic loading, long-term durability, sterilization compatibility, and hemocompatibility—cannot be drawn from the current data alone. Therefore, the results should be interpreted primarily as a mechanical characterization and comparative assessment of FDM-printed TPU and SEBS, rather than as a validation of device-level performance. Future studies integrating physiological testing conditions and comprehensive biocompatibility evaluation are essential before these materials can be considered for clinical translation.

### 4.6 Concluding remarks

The results of this study provide the first systematic characterization of the mechanical properties of FDM-printed TPU and SEBS aortic valve leaflets, addressing a critical gap in the literature. The findings demonstrate that both materials are viable candidates for polymeric TAVI devices, with TPU offering greater compliance and ductility, and SEBS providing higher stiffness. The anisotropic behavior arising from the FDM process must be carefully considered in both the design and numerical simulation of these devices. The proposed experimental and numerical approach can help manufacturers in the elaboration and manufacturing of new TAVI polymer devices made of TPU and SEBS polymers for clinical applications. Future research should focus on fatigue testing, biocompatibility assessment, and the development of accurate constitutive models to support the translation of these materials into clinically viable polymeric heart valves.

## Data Availability

The original contributions presented in the study are included in the article/Supplementary Material, further inquiries can be directed to the corresponding author.
