# Supplementary material for: Experimental study of TPU and SEBS additively manufactured leaflets for novel aortic valve polymer TAVI prosthesis
Source: Front Bioeng Biotechnol. 2026 Oct 21;14:1959014. doi: 10.3389/fbioe.2026.1959014 (PMC13731260; doi:10.3389/fbioe.2026.1959014)
Supplement: Supplementary file 1 [file Supplementaryfile1.docx]

**Workflow in ANSYS Workbench**

**Step 1: Create a Static Structural Analysis Project**

1. Launch ANSYS Workbench.
2. Drag and drop a Static Structural analysis system from the Toolbox into the Project Schematic.
3. Double‑click on Engineering Data to open the material editor.

**Step 2: Define a New Hyperelastic Material**

1. In the Engineering Data workspace, click Click here to add a new material and name it (e.g., *TPU_MooneyRivlin_5P* or *SEBS_MooneyRivlin_2P*).
2. Ensure the unit system is consistent with your experimental data (e.g., MPa for stress).

**Step 3: Import Experimental Test Data**

ANSYS requires experimental data in the form of engineering stress vs. engineering strain from one or more deformation modes. For the present study, uniaxial tensile test data (from Section 2.4) are the primary input.

1. In the Toolbox on the left, expand Hyperelastic Experimental Data.
2. Drag Uniaxial Test Data into the Properties window of your new material.
3. A table will appear. Enter your experimental stress–strain pairs (engineering stress in MPa, engineering strain as a decimal, e.g., 0.1 for 10% strain). Alternatively, you can import data from an external file (CSV or TXT).

Note: For more accurate calibration, it is recommended to also include Biaxial Test Data and Shear Test Data if available. However, uniaxial data alone can provide reasonable parameter estimates, especially when the material is primarily loaded in tension, as in valve leaflets.

4. Repeat the process for Biaxial Test Data and Shear Test Data if such experiments were performed.

**Step 4: Select the Mooney‑Rivlin Model**

1. In the Toolbox, expand Hyperelastic.
2. Drag Mooney‑Rivlin 5 Parameter into the Properties window for TPU, or Mooney‑Rivlin 2 Parameter for SEBS.
3. The model will appear in the Outline tree under your material.

**Step 5: Perform Curve Fitting**

1. In the Outline tree, right‑click on Curve Fitting (which appears automatically after adding the hyperelastic model) and select Solve Curve Fit.
2. ANSYS will perform a least‑squares minimisation to determine the material constants that best match the experimental data.
3. The calculated coefficients will be displayed in the Properties window.

Tip: If the fit is poor, check the quality of your experimental data (e.g., remove outliers, ensure smooth curves) or consider using a different hyperelastic model (e.g., Ogden or Yeoh). The Mooney‑Rivlin model works well for strains up to approximately 100–200%.

**Step 6: Copy Calculated Values to Material Properties**

1. Right‑click on Curve Fitting again and select Copy Calculated Values to Property.
2. The optimised coefficients will now be permanently assigned to the material constants.

**Step 7: Set the Incompressibility Parameter**

For nearly incompressible materials such as TPU and SEBS, the parameter *d* should be set to a small value, which enforces near‑incompressibility without causing numerical issues.

**Step 8: Validate the Fit**

1. In the Curve Fitting details, you can plot the fitted curve against the experimental data points to visually assess the quality of the fit.
2. If the fit is unsatisfactory, consider:
   - Using a higher‑order model,
   - Including additional deformation modes (biaxial, shear) in the calibration,
   - Adjusting the initial guesses for the coefficients (in advanced settings).
