# Supplementary material for: Experimental study of TPU and SEBS additively manufactured leaflets for novel aortic valve polymer TAVI prosthesis
Source: Front Bioeng Biotechnol. 2026 Oct 21;14:1959014. doi: 10.3389/fbioe.2026.1959014 (PMC13731260; doi:10.3389/fbioe.2026.1959014)
Supplement: Supplementary file 2 [file Supplementaryfile2.docx]

### **Supplementary material 2**

### **Mathematical formulation of TPU/SEBS leaflet compression**

For the numerical experiment, a hyperelastic nonlinear material model for TPU and SEBS (Section 2.8) based on the processing of compression test data was used. The geometry of the numerical test model corresponded as closely as possible to that used in the physical experiment. The valve model was positioned vertically between a fixed lower plate and a movable upper plate. The upper plate moved downward until a displacement of 10 mm was reached, thereby creating pressure on the leaflets. The geometry of the problem is shown in Fig. S1.

Fig. S1. Problem formulation

Equilibrium equation

| $\sigma_{ij,j}=0,x\in\Omega_{n},n=\overrightarrow{1,3},$ | (1) |
| --- | --- |

$\sigma_{s}=\frac{\partial W}{\partial\varepsilon},$	(2)

The Mooney‑Rivlin strain energy potential is expressed as a polynomial function of the first and second invariants of the right Cauchy‑Green deformation tensor

| $W=C_{10}\left( I_{1}-3 \right)+C_{01}\left( I_{2}-3 \right)+{C_{20}\left( I_{1}-3 \right)}^{2}+C_{11}\left( I_{1}-3 \right)\left( I_{2}-3 \right)+{C_{02}\left( I_{2}-3 \right)}^{2}+\frac{1}{d}\left( J-1 \right)^{2}$, | (3) |
| --- | --- |

where *W* is the strain energy density, *I*_1_ and *I*_2_ are the first and second invariants of the right Cauchy‑Green deformation tensor, *C*_10_, *C*_01_, *C*_20_ *C*_11_ *C*_02_ are material constants, *d* is the incompressibility parameter (typically set to a small value for nearly incompressible behaviour), *J* is the Jacobian determinant of the deformation gradient.

For the 2‑parameter model (used for SEBS), the higher‑order terms (*C*_20_, *C*_11_, *C*_02_) are omitted, and the potential reduces to:

| $W=C_{10}\left( I_{1}-3 \right)+C_{01}\left( I_{2}-3 \right)+\frac{1}{d}\left( J-1 \right)^{2}.$ | (4) |
| --- | --- |

Geometry relations:

$\varepsilon=\frac{1}{2}\left( \nabla u_{s}+{(\nabla u_{s})}^{T}+(\nabla u_{s})\boldsymbol{\cdot}{(\nabla u_{s})}^{T} \right),$	(5)

where $u_{s}$ is the displacement field, $\varepsilon$represents the Green–Lagrange strain tensor.

Boundary conditions

| $u_{x,y,z}=0,\overrightarrow{x}\in S_{5}$ | (6) |
| --- | --- |
| ${u^{'}}_{x,z}=0, {u^{'}}_{y}=1$mm/min, $\overrightarrow{x}\in S_{1}$ | (7) |
| $\sigma_{ij}\cdot n_{i}=0, \vec{x}\in S_{l},$ | (8) |

Contact

*– Bonded contact for bodies* $\Omega_{n},n=\overrightarrow{1,2},$at $S_{2}$

| $\left\Vert\text{u}_{i}\right\Vert=0,\left\Vert\sigma_{ij,j}\cdot\text{n}_{i}\right\Vert=0$ | (9) |
| --- | --- |

*– Contact with friction on surfaces* $\Omega_{n},n=1,3$ at $S_{4}$
$$F_{friction}=\mu\cdot N,\mu=0.2$$

(10)
